# Polymers Based on PLA from Synthesis Using D,L-Lactic Acid (or Racemic Lactide) and Some Biomedical Applications: A Short Review

**DOI:** 10.3390/polym14122317

**Published:** 2022-06-08

**Authors:** Juliene Oliveira Campos de França, Deborah da Silva Valadares, Mateus Freitas Paiva, Sílvia Cláudia Loureiro Dias, José Alves Dias

**Affiliations:** Laboratório de Catálise, Instituto de Química, Campus Universitário Darcy Ribeiro—Asa Norte, Universidade de Brasília, Brasília 70910-900, Brazil; julienechemistry@gmail.com (J.O.C.d.F.); deborahsvaladares@gmail.com (D.d.S.V.); freitas-paiva@hotmail.com (M.F.P.)

**Keywords:** poly(lactic acid)-PLA, polymerization based on D,L-lactic acid, D,L-lactide, stereo polymerization of D,L-lactic acid, blends of PLA, poly(DL-lactide-co-polymer), composites of PDLLA

## Abstract

Poly(lactic acid) (PLA) is an important polymer that is based on renewable biomass resources. Because of environmental issues, more renewable sources for polymers synthesis have been sought for industrial purposes. In this sense, cheaper monomers should be used to facilitate better utilization of less valuable chemicals and therefore granting more sustainable processes. Some points are raised about the need to study the total degradability of any PLA, which may require specific composting conditions (e.g., temperature, type of microorganism, adequate humidity and aerobic environment). Polymerization processes to produce PLA are presented with an emphasis on D,L-lactic acid (or rac-lactide) as the reactant monomer. The syntheses involving homogeneous and heterogeneous catalytic processes to produce poly(D,L-Lactic acid) (PDLLA) are also addressed. Additionally, the production of blends, copolymers, and composites with PDLLA are also presented exemplifying different preparation methods. Some general applications of these materials mostly dedicated to the biomedical area over the last 10–15 years will be pointed out.

## 1. Introduction

There is a vast contribution of polymers to the chemical industry development since the beginning of the twentieth century. These macromolecules are characterized by their properties such as size, structure, and intra/intermolecular interactions. Polymers are widely used in modern society, and some can be considered feedstock for the fabrication of a variety of other materials. Because there is huge disposal of polymer waste in all countries, a considerable effort has been put to control this process and a primary concern is to seek a solution. In this sense, bio-renewable polymers made from sustainable supplies are poised as a promising solution, even though the substitution of traditional polymers obtained from petroleum might not be a permanent answer yet.

Among different polymers, poly(lactic acid) (PLA) is based on renewable resources and could be employed, in principle, in sustainable plastics. In a circular economy, it could be considered a promising biopolymer due to its properties and estimated commercial expenses. In order to increase the attractiveness of PLA, the use of cheaper sources of monomers (e.g., D,L-lactic acid) should be considered. The relatively low attention to the use of D,L-lactic acid as a source for PLA may be explained by the predominant creation of amorphous materials. However, there are a number of polymers and mainly copolymers based on PLA that are based on D,L-lactic acid in some of the steps of preparation. Thus, this brief review attempts to show some of these polymers described in the literature and their respective applicability, mostly as relates to the biomedical area. A brief discussion on the general sustainability of PLA and some of its fundamental characteristics is also presented.

## 2. A Few Points about the Problem of Plastics

The dependence of today’s society on plastic materials is indisputable. The low production cost linked to the various functions they can perform makes them extremely advantageous for various applications. In the mid-twentieth century, synthetic plastics began to be ostensibly used in the textile, automotive, electronics, food, packaging, and various other segments, bringing great technological advances and considerable benefits to humanity [1,2]. On the other hand, the exacerbated consumption of these plastic materials, especially commodity polymers (typically from fossil sources), can lead to the accumulation of their waste in the environment. Because they are not significantly biodegradable and are susceptible to photodegradation, these plastics can be fragmented into smaller particles of micro and even nanometer dimensions and can cause several environmental problems [3]. The presence of these particulate materials in aquatic environments such as freshwater systems [4], oceans and seas [5,6,7,8,9,10,11] worries the scientific community and society, as they can cause an imbalance in these ecosystems, for example, compromising the photosynthetic capacity of algae and plankton. Another recurring concern has arisen regarding the ingestion of microplastics by human beings, often from contaminated marine products [12,13] and even through the airways [14,15], which can cause potential health risks.

The excessive production and consumption of non-degradable plastics can lead to the aggravation of global warming due to a series of reasons: the emission of greenhouse gases during the production process and after the end of its useful life [14]; pollution of aquatic and terrestrial environments due to improper disposal and treatment of waste [16]; long periods of decomposition (which can reach thousands of years), and low recycling rate [17]. The decomposition time of some plastic waste can be seen in the recent review by Chen et al. [17].

Plastics are considered non-toxic materials, being neither digested nor absorbed in the intestine [18]. However, during processing and manufacturing, additives are added to the plastic resin such as flame retardants and plasticizers, for example. Some of the main additives used in plastic packaging, such as bisphenol A, polybrominated diphenyl ether (PBDE) and phthalates are potential endocrine disruptors [19,20,21]. Studies show that these additives can migrate from packaging to food and bottled water [22,23] even in low amounts, raising an alert about their impact on human health. A viable alternative for many of these problems is the use of sustainable, bio-based and/or biodegradable polymers. The monomers that give rise to these polymers can be obtained from first or second-generation biomass. Despite the advantages associated with the use of bio-based polymers, such as reduced carbon dioxide emissions and dependence on fossil resources, there are some disadvantages related mainly to the use of first-generation biomass in the production of inputs for biofuels and bioplastics, such as competition with food and feed supplies, deforestation, excessive use of pesticides and fertilizers, and consumption of water for irrigation [24,25]. In addition, it is common during large-scale industrial production of bio-based polymers and biopolymers to make use of metallic catalysts, which can remain in the finished product causing environmental problems or even being toxic to the end user. This can occur, for example, in the industrial production of PLA, in which tin, zinc or aluminum-based catalysts are often required, and may have a carcinogenic effect [26]. However, a path already explored, although more expensive, could be the use of second-generation biomass, food residues, microalgae, and others, and, still, use of metal-free catalysts such as organic or enzymatic catalysts.

**Figure 1:**
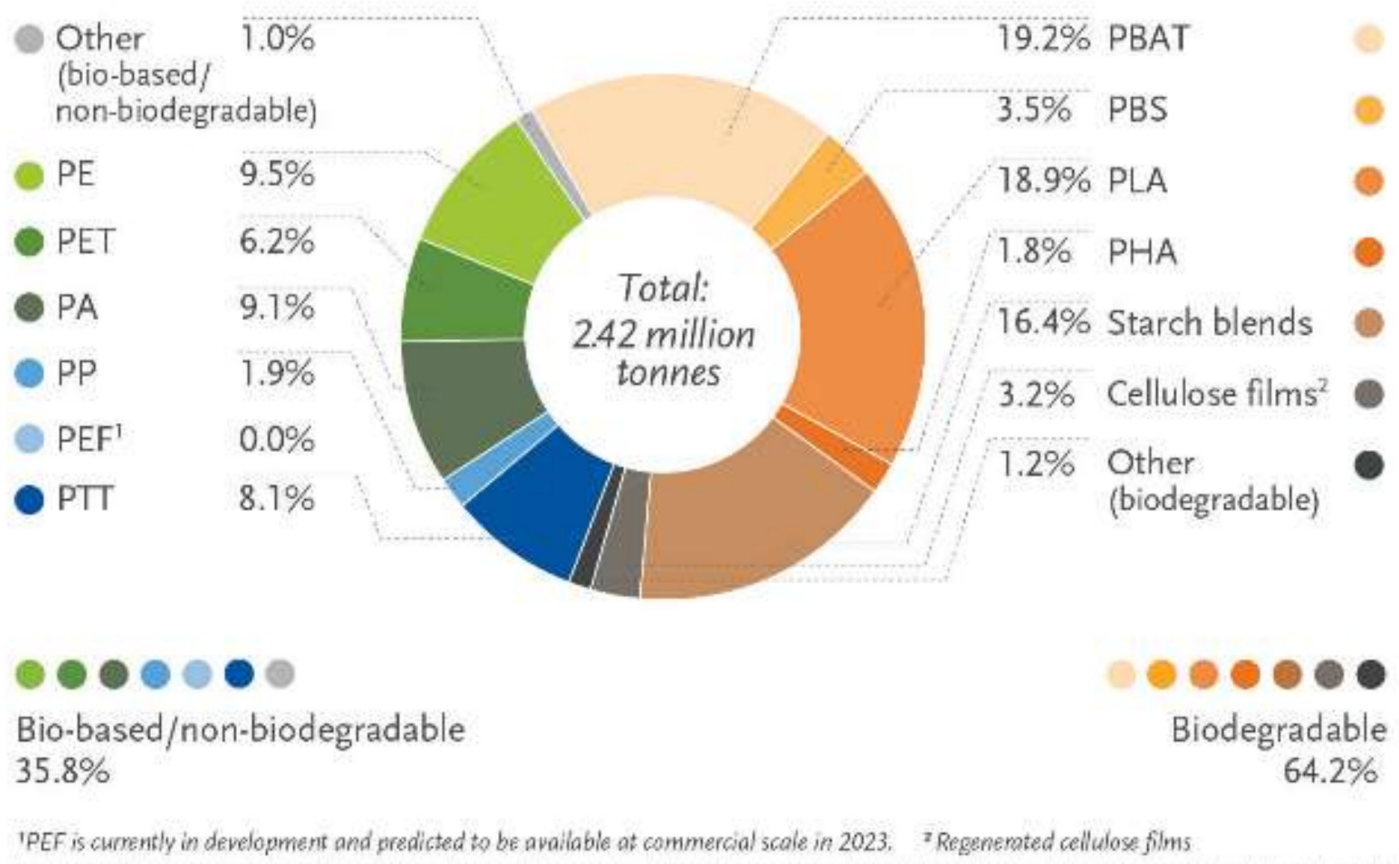
The global production of bioplastics by material types. Copyright European Bioplastics Nova-Institute (2021). Available online: https://www.european-bioplastics.org/news/publications/#MarketData (accessed on 1 June 2022).

According to the report carried out by European Bioplastics in collaboration with the Nova-Institute on the prospects for the production of bioplastics (a category that includes bio-based and/or biodegradable polymers) in the period from 2021 to 2026, approximately 367 million tons of plastics will be produced in the world annually, of which less than 1% corresponds to bioplastics [27]. In 2021, about 50% of the global production capacity of bioplastics was destined for the area of rigid and flexible packaging, as can be seen in Figure 1, described in [17]. Even though the packaging segment is currently the main one in terms of application of these polymers [28], others tend to increase their use, due to the possibility of modeling their properties from functionalization, copolymerization, blending with other polymers, the addition of plasticizers and additives [29,30].

## 3. Monomers of Lactic Acid and Lactides to Produce PLA

**Figure 2:**
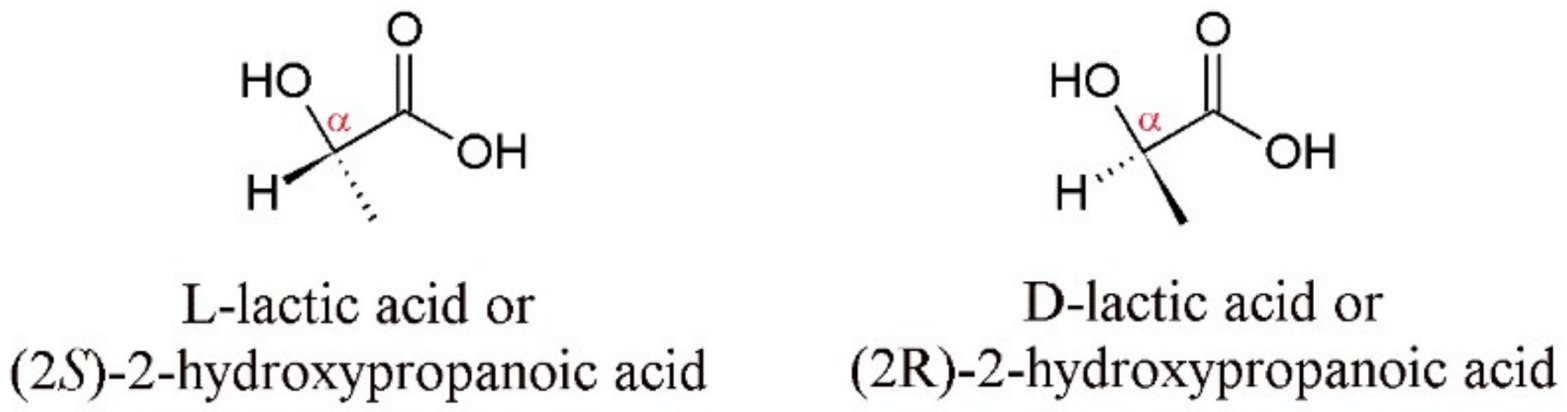
Lactic acid enantiomers. Common nomenclature and IUPAC nomenclature of the optical isomers of lactic acid. The α-carbon characteristic of α-hydroxycarboxylic acids is highlighted in red.

One of the main bio-based polymers currently produced is PLA—polylactide or poly(lactic acid)—which is a lactic acid-based polymer that stands out as a potential replacement for some highly consumed thermoplastic polymers, such as PET (polyethylene terephthalate) and PS (polystyrene) [31,32,33]. The PLA building block is lactic acid and lactide. Lactic acid (2-hydroxypropanoic acid) is one of the simplest chiral organic molecules known. It was discovered in 1780 by the Swedish chemist Scheele, and it can be produced chemically or biologically, in two isomeric forms: D-lactic acid (R, (−)-lactic acid) and L-lactic acid (S, (+)-lactic acid, in addition to the racemic mixture, commonly called D,L-lactic acid [34,35,36]. It belongs to the class of α-hydroxy acids, which are weak organic acids with one or more hydroxyl groups attached to the alpha carbon, which is the first after the acid group [37,38], as shown in Figure 2. Lactic acid, as well as many other hydroxy acids, is often in high demand in pharmaceutical, food, and biomedical applications, acting as a food additive, preservative, acidulant, cosmetic, personal care product and the building block of bio-based polymers [35,39,40], in addition to playing a vital role in the glycolytic energy cycle [29,36,41]. The bifunctionality of lactic acid, conferred by the alcohol and carboxylic acid groups, makes this molecule susceptible to different reactions, such as condensation, reduction, esterification, and substitution in the alcohol group, and considerably expands its applications [35].

As previously mentioned, lactic acid (LA) can be obtained from chemical or biological synthesis. Chemical synthesis exclusively generates the racemic mixture of lactic acid (D,L-lactic acid), from a reaction between hydrogen cyanide (HCN) and acetaldehyde (CH_3_CHO) at high pressures and in the presence of a base. From this reaction, lactonitrile (CH_3_CHOHCN) is obtained, which is hydrolyzed by the addition of sulfuric or hydrochloric acid, resulting in LA and ammonium sulfate or ammonium chloride as a by-product [42]. The lactic acid produced in this step is then esterified with methanol, generating the methyl lactate ester, which is purified and recovered by distillation, and then hydrolyzed in an aqueous medium, producing lactic acid and methanol. Methanol can be recovered and recycled at the end of the process [41,42,43,44]. There are also two other more expensive and rarely used methods of chemical synthesis of LA: (i) it consists basically of using acrylonitrile instead of lactonitrile in the method described above; (ii) it uses propionic acid as a raw material for the LA production [29]. Catalytic procedures can be used to obtain D,L-lactic acid from raw materials such as vinyl acetate or glycerol [42]. Some of the main disadvantages of the chemical synthesis of LA are the dependence on raw material of petrochemical origin. Some key questions are the high cost associated with variations in the price of oil, resource availability and the exclusive obtention of the racemic mixture of LA as a product. D,L-LA does not meet the optical purity requirements for most applications that are intended (e.g., in the food, beverage and pharmaceutical industry) because of the metabolic problems that D-LA can cause [40,43,44]. Despite this, there is a company, Musashino Japan, that still produces D,L-lactic acid and its esters by chemical synthesis, in addition to some Chinese manufacturers that produce the racemic mixture by fermentation, using non-specific microbes [45].

**Scheme 1:**
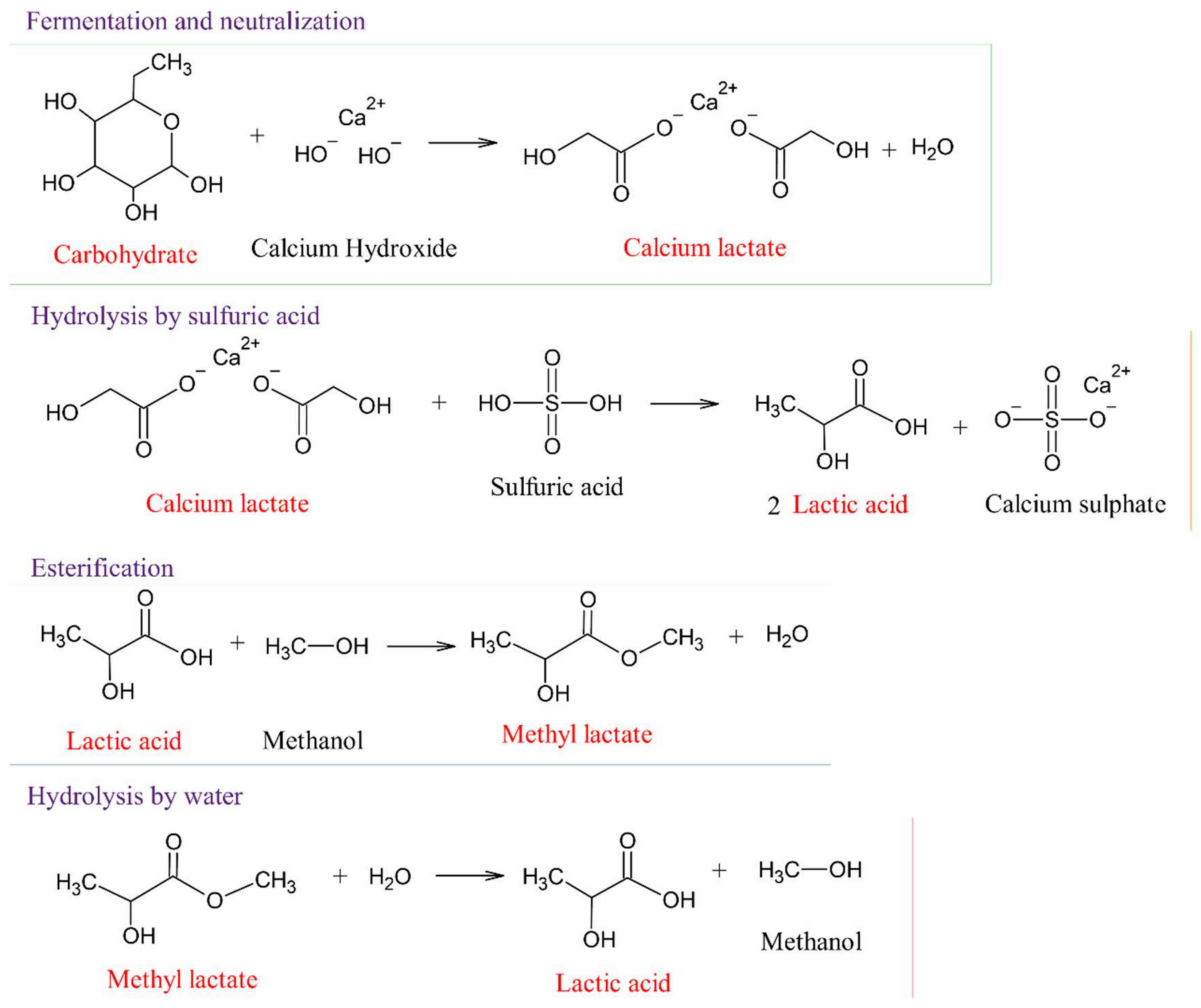
Fermentation procedure for obtaining LA.

Currently, about 90% of the lactic acid marketed worldwide is obtained via microbial fermentation of first-generation biomass sugars, such as corn starch and sugarcane broth [34,46]. These agricultural products are converted into lactic acid by the action of lactic acid bacteria (LAB) that can produce the enantiopure L-lactic and D-lactic acid, or even D,L-lactic acid, as is the case with *Lactobacillus helveticus* [32,42]. Presently, all commercialized processes for the production of lactic acid depend on refined carbohydrates, such as glucose, lactose-containing whey and sucrose from sugarcane and beetroot [45,46]. The use of these refined carbohydrates, however, results in higher production costs, motivating the search for lower-cost substrates such as lignocellulosic biomass, seaweeds, domestic organic waste (e.g., food waste) and residues from the dairy industries [47,48,49,50,51,52]. Microbial fermentation of carbohydrates is more economically viable than chemical synthesis, with carbohydrates being converted to lactic acid in batch reactors using water and bacterial cultures in a broth-like mixture. This broth, having calcium lactate and other impurities, undergoes filtration, carbon treatment, evaporation and acidification with sulfuric acid to remove cells and obtain lactic acid and calcium sulfate. As calcium sulfate is insoluble, it is removed by filtration, and lactic acid is hydrolyzed, esterified, distilled and hydrolyzed [42]. Scheme 1 shows the sequence of the main steps involved in a conventional lactic acid fermentation process, as described in literature [53].

**Scheme 2:**
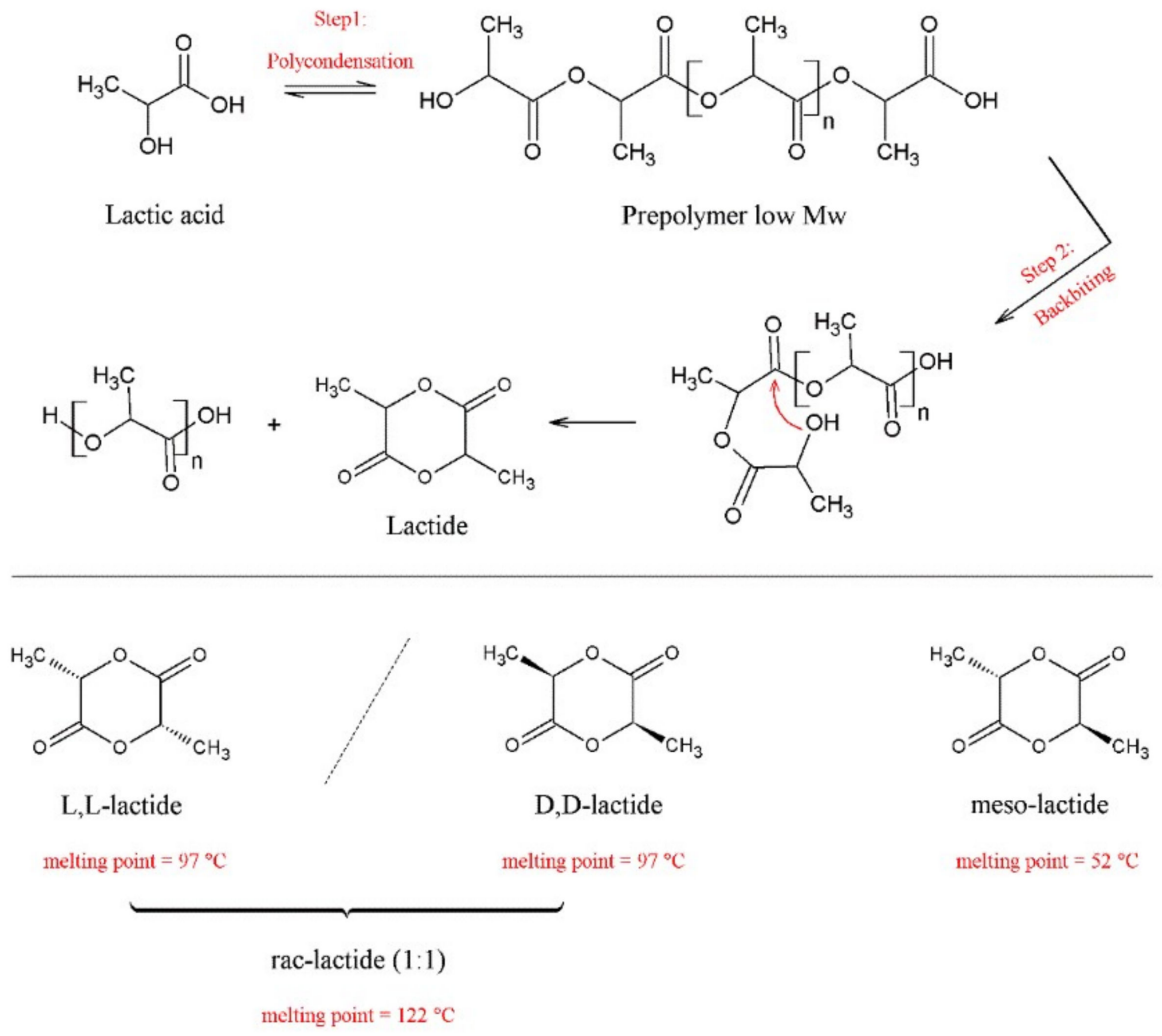
Two-step process for the synthesis of lactide and its optical isomers.

Lactide is the cyclic dimer of lactic acid and the most important building block in the production of PLA [46,54]. The lactide has two stereocenters, which can both have the same configuration, in the case of L,L-lactide and D,D-lactide, or both with different configurations, in the case of meso-lactide. In addition to these diastereomers, there is also the possibility of obtaining racemic lactide (rac-lactide), which consists of a 1:1 mixture of L,L-lactide and D,D-lactide [32,54,55,56,57,58]. The melting point of meso-lactide is significantly lower than that of enantiopure lactides and rac-lactide (122, 97 and 52 °C, respectively), which makes it possible to isolate it from the other isomers by distillation or crystallization techniques [41,44,54]. The conventional synthesis of lactide takes place in a two-step process that consists of the polymerization of lactic acid by polycondensation and depolymerization of this polymeric species to obtain the lactide (Scheme 2). The polycondensation step is usually carried out under vacuum and at high temperature to ensure the removal of water from the system and the condensation of lactic acid molecules, leading to the formation of short-chain oligomers and low relative molar mass (<5 kDa). The backbiting step, which is an endothermic transesterification reaction [59,60,61], occurs in the presence of a metallic catalyst, normally a soluble salt based on tin, zinc, antimony, aluminum, etc. forming oligomers and lactide under reduced pressure [32,41]. As lactide is formed together with oligomeric fractions of lactic acid, it needs to be continuously removed from the reaction medium during synthesis, through successive distillation techniques using, for example, azeotropic solvents which makes the process even more expensive [58]. It is estimated that about 30% of the total cost associated with the production of PLA comes from the synthesis of lactide [54].

Because of the high cost associated with the two-step synthesis of lactide dimer, many studies have been carried out to develop more economical and viable processes to obtain this important substrate. The main reported processes in the literature involve: (i) the synthesis of lactide in the gas phase under a fixed bed of solid catalysts, such as aluminum oxide, silica nanocomposites, and silica-alumina [54,61,62,63]; (ii) synthesis of lactide in the one-step liquid phase, directly from lactic acid, using azeotropic solvents (e.g., toluene; xylenes) and zeolitic catalysts with potential shape selectivity and other porous solids [60,64,65]. The above-mentioned processes take place in the presence of heterogeneous catalysts, instead of the classic catalysts based on soluble salts of Sn, Zn and Al. Some benefits include (i) operability at ambient pressure; (ii) more energy efficient than the conventional two-step process; (iii) low index of product racemization; (iv) high selectivity and high lactide yields [42,54]. Some problems to be overcome are replacing toluene with sustainable solvents in liquid phase synthesis (e.g., xylenes); optimizing the reaction parameters in the gas phase to increase the lactide volumetric productivity (which is much lower than that of the industrial process) [54]. Thus, these processes could become even more suitable for the industrial production of lactide, consequently reducing the cost associated with the production of PLA.

## 4. Poly(lactic acid) (PLA) Polymers

PLA is one of the main bio-based polymers sold worldwide. It is considered a hydrolytically degradable, compostable, biocompatible and bioabsorbable polymer, which makes it attractive for applications in the biomedical area (tissue engineering, drug delivery system, sutures, etc.), in agriculture, ecology, packaging, among others [66,67]. It is a thermoplastic polyester that has a processing temperature in the range of 170 to 230 °C, and can be processed by extrusion, spinning, biaxial stretching and blown injection, thus covering a wide and dynamic field of applications, including food packaging, textile, and non-textile fabrics (such as curtains and wet wipes, respectively), toys, among others [29,41,68]. When mixed with other polymers or in the form of composites, some properties of PLA can be modified and improved, such as flexibility, impact resistance and heat stability, allowing its application as flexible films and engineering plastics [41]. Nevertheless, despite being often classified as a biodegradable plastic, PLA, especially the high molar mass (>100 kDa), is hardly biodegradable under ambient conditions, requiring very specific composting conditions (e.g., temperature above Tg (glass transition temperature), specific microorganism, adequate humidity, and aerobic environment for its complete decomposition into CO_2_ and H_2_O) [25,67].

### 4.1. A Brief Outline

Poly(lactic acid) or polylactide (PLA) is a bio-based aliphatic polyester produced from renewable resources such as wheat, straw, corn, cellulose, starch, sorghum etc., [29,36,42]. In the early 1800s, Pelouze used a lactic acid distillation process to remove water (polycondensation), thus obtaining a low molar mass PLA [69]. Later, in 1932, Wallace Carothers, a DuPont scientist, synthesized PLA by heating lactide under a vacuum, in a process known as ROP (Ring-Opening Polymerization) [57,69,70,71]. The PLA obtained so far had characteristics that limited its potential for application, such as low molar mass and instability in a humid atmosphere [71]. In 1954, a lactide purification method was developed by DuPont and made it possible to obtain high molar mass (HMW) PLA [70,72]. However, it was from 1966 onwards, with the studies by Kulkarni et al., about the biodegradation and non-toxicity of PLA [71,73] that it and its copolymers started to be applied in the biomedical field as sutures, prostheses, matrices for drug delivery systems, scaffolds among others [31,42,69,71,72].

The advancement and dissemination of PLA production and processing technology have caused its field of applications to expand significantly in recent decades, especially after the creation, in 2002, of the NatureWorks (USA) industrial large-scale PLA production plant, which currently operates with a production capacity of 150,000 metric tons/year [41,69,74,75]. The joint venture Total and Corbion (Amsterdam, The Netherlands) and Hisun (Wuhan, China) also produce PLA on a large scale, with a production capacity of 75,000 and 10,000 metric tons/year, respectively [41,42,69,75,76]. Other manufacturers produce PLA on a smaller scale and have been listed in the references [32,41,77].

### 4.2. Synthetic Routes

High molar mass PLA can be obtained from three main synthetic routes: direct condensation polymerization, azeotropic dehydration condensation and lactide ring-opening polymerization (ROP) [55].

Direct polycondensation takes place from the dehydration of lactic acid with simultaneous esterification of the monomers and the release of water for each acid unit added by condensation [78]. Water readily reacts with the formed oligomers, shifting the equilibrium towards the reactants and making it difficult to obtain a high molar mass polymer [41,45,59,79]. Removing condensed water from the reaction medium is quite complicated, as the increase in the concentration of oligomers leads to an increase in the viscosity of the medium [41,42,45]. This requires the use of high temperatures, in the range of 150–200 °C, pressure below 5 torr and a long reaction time in the presence of a chain coupling agent [29,31,32,55,70,80,81], or in some cases, an azeotropic solvent [32]. Other alternatives for the synthesis of high molar mass PLA by polycondensation such as solid-state polycondensation (SSP) were discussed by Masutani [32].

In condensation by azeotropic dehydration, first the LA is distilled under a vacuum for about 3 h to remove most of the condensation water. Then, the azeotropic solvent (diphenyl ether) and the catalyst are added to the LA solution, with the solvent being refluxed and returning to the reaction flask after passing through a molecular sieve, without the need for chain extenders or adjuvants to obtain high molar mass PLA [29,31,32,42,55,70]. The main disadvantages of this synthesis lie in the use of diols and diacids as solvents, catalyst residue and low yields [29,78].

The most widespread and industrially employed route in PLA synthesis is lactide ring-opening polymerization (ROP). ROP is a propagation process of cyclic monomers initiated by different ions [78] and generally occurs in a two-step process. The first step consists of obtaining lactide with high optical purity (generally by the two-step process mentioned in Section 3); the second consists of the lactide polymerization promoted by an initiator or catalyst [31,55,70,82]. The catalysts commonly used in this synthesis are metallic catalysts, such as Zn and Sn oxides, zinc and tin chlorides or tin octoate [78,82,83,84].

Compared to direct polycondensation, ROP can be performed under milder conditions, such as a reaction temperature of 130 °C and a shorter reaction time [32,78,85]. ROP can be classified in terms of the reaction mechanism as: anionic polymerization, cationic polymerization, and coordination-insertion mechanism [32,55,78,80,85,86]. The most popular catalyst used in this synthesis is tin(II) bis-2-ethylhexanoic acid (tin octoate), due to its solubility in molten lactide, low product racemization, high conversion and catalytic activity, and for providing PLA of high molar mass (≥1000 kDa) [72,80,84]. Another relevant aspect of ROP is that it makes it possible to control the microstructure of the polymer, including the order of insertion of monomers in the polymer chain based on their stereochemistry, as well as the combination of reaction time, temperature, type, and concentration of catalyst [80,82,86].

### 4.3. Structural Variety and PLA Properties

PLA presents a great structural diversity based on its enantiomeric constitution. The enantiopure monomers of L-lactic acid and D-lactic acid (or their lactide analogues) lead to the formation of poly(L-lactic acid) (PLLA) and poly(D-lactic acid) (PDLA), respectively. These homopolymers are semi-crystalline and have the same thermal properties, such as melting temperature (T_m_) ranging between 170–180 °C, glass transition temperature (T_g_) around 55–60 °C and crystallinity around 35% [29,31,32,82,84,87,88]. PLLA with a percentage of L-isomer above 90% in its composition tends to be semi-crystalline, while PLA with a content lower than this tends to be amorphous [89].

Polymerization of the racemic mixture of lactic acid, rac-lactide (rac-LA) and meso-lactide (m-LA) results in poly(D,L-lactic acid) or PDLLA, a random copolymer of D and L-lactic units, with an irregular and completely amorphous structure [71,75,90]. PDLLA has no T_m_ but has a T_g_ around 60 °C [32,87]. Due to its amorphous nature, PDLLA shows a faster degradation rate than stereoregular PLA, making it preferred for applications as a drug delivery vehicle and as a low-strength scaffolding material for tissue engineering [75,91]. PDLLA may show some crystallinity when synthesized by stereocontrolled ROP, through the action of a catalyst/initiator [32,80]. Aluminum alkoxide catalysts, Schiff bases and other single-site complexes have been used in the synthesis of stereoregular PDLLA [80].

**Scheme 3:**
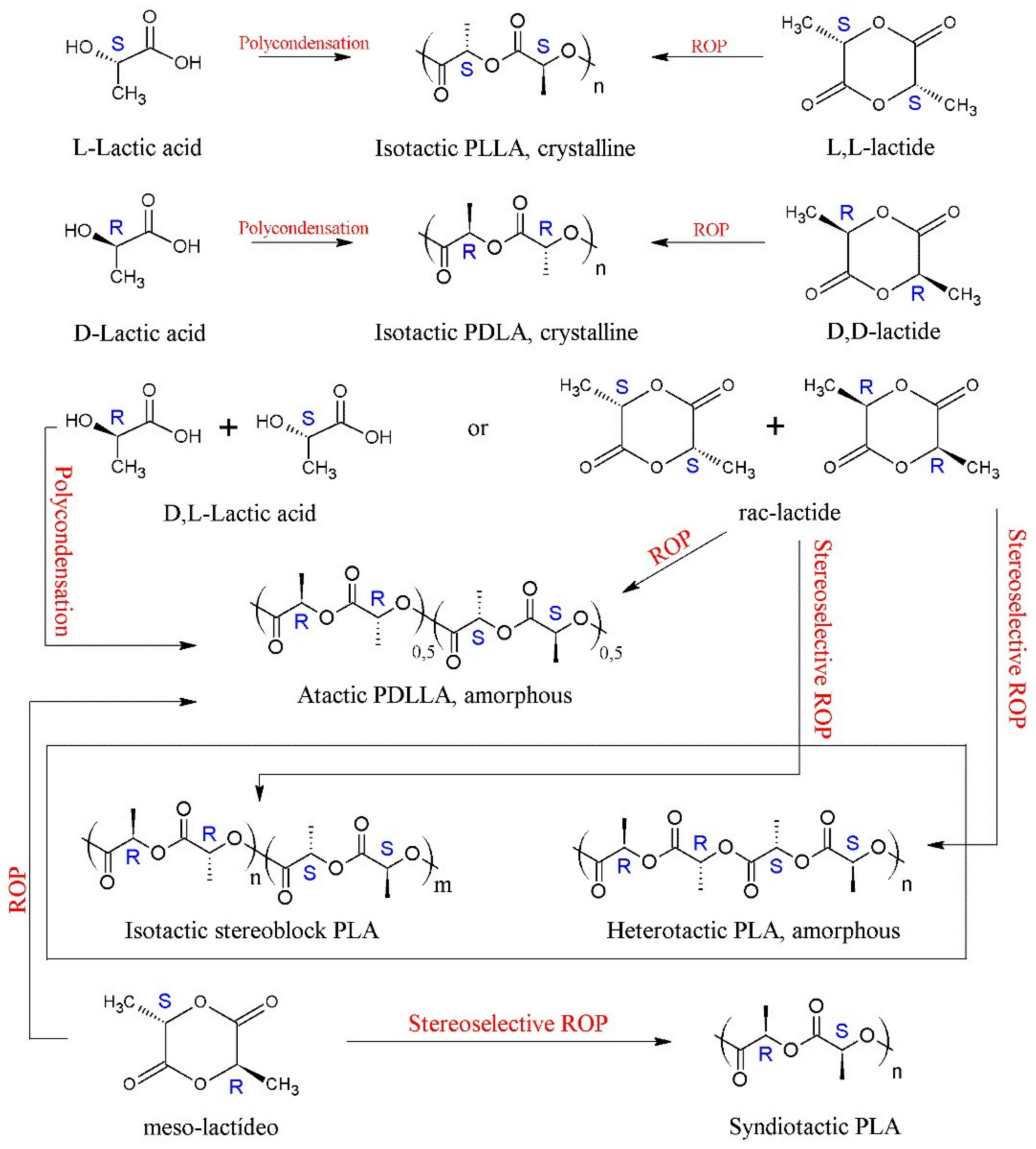
The variety of PLA microstructures and synthetic routes.

In 1987, Ikada et al., reported, for the first time, that a blend of PLLA and PDLA in equal proportions (1:1) produced stereocomplex crystals, which had different properties from pure homopolymers [32,92]. PLA stereocomplexes (sc) have a melting temperature (T_m_) about 50 °C higher than that of homopolymers, varying around 230 °C [93,94]. However, these sc-PLA can be formed concomitantly with PLA homocrystals (hc), as the kinetics of homocrystal formation is favored in high molar mass PLLA/PDLA mixtures (weight-average molecular weight, Mw > 40 kDa) [95], thus limiting the exclusive production of sc-PLA. One way to circumvent this problem is the production of stereoblock PLA (sb-PLA), which is a copolymer containing isotactic sequences of PLLA and PDLA [96]. The sc-PLA and sb-PLA can be obtained from the stereoselective ROP (rac- or m-LA) using a chiral catalyst [97,98], as can be seen in Scheme 3, which has been described in the literature [32]. In addition to stereoblock synthesis, other alternatives to improve PLA stereocomplex are melt blending, the addition of nucleating agents and polymer blending [93]. Several review articles have been published on the synthesis, structure, crystallization behavior, other properties, and applications of these sc-PLA [99,100,101,102,103].

### 4.4. PLA Modifications: Blends, Copolymers and Composites

Some common desired properties of PLA, such as rigidity, permeability, crystallinity and thermal stability, and hydrophobicity/hydrophilicity can be improved by processes such as copolymerization, blending and production of polymer composites [36]. The copolymerization process consists of the simultaneous polymerization of two or more monomers that interact via chemical reactions and produce PLA copolymers. These copolymers can be sequenced alternately, in blocks or grafts, but this considerably compromises their biocompatibility. However, biocompatibility can be improved by the copolymerization of lactic acid with other hydrophilic monomers or polymers. For example, block copolymers of lactic acid and polyethylene glycol (PEG) are hydrophilic and some of them are even water-soluble [100].

The blending process consists of two or more different polymers mechanically mixed and connected through physical interactions [104], producing blends. That is the case of PLLA/PDLA, PLLA/PDLLA, PLA/starch blends, PLA/PHB (polyhydroxybutyrate) and others. In the literature [88,105,106] several possibilities of PLA blends were revised, considering systems that include mixtures with hydrophobic and hydrophilic polymers, other polyesters and so on. A recent review article by Hamad et al., discussed the production of PLA modified by polymer blending techniques to achieve suitable properties for some applications, such as medical, packaging, battery and semiconducting [107]. They pointed out that biodegradable polymers have been mostly utilized due to their environmental advantages and high toughness compared with pure PLA, but other polymers still show low cost, better mechanical properties, high thermal stability, and processability. For instance, they verified in the literature that PLA/TPS (PLA-thermoplastic starch) blend could be useful in general packaging applications by means of compatibilizers such as PLA-g-MA (PLA-g-maleic anhydride), GMA-g-PEO (glycidyl methacrylate-g-poly(ethylene oxide)), TPS-g-MA (thermoplastic starch-g-maleic anhydride), PLA-g-TPS (PLA-g-thermoplastic starch). The PLA blend improved the biodegradation rate, but this process promoted weakness and low elastic modulus. Some PLA blends also have a higher flexibility than pure PLA, which make this material more appropriate for the fabrication of 3D scaffolds. Furthermore, PLA blends can be used to produce hierarchically porous materials for biomedical applications, since micropores can enhance the tissue ingrowth and the smaller pores (submicrometer scale) can provide the cell differentiation. Given this scenario, the authors proposed that research on PLA blends should preferably focus on their application. In addition, significant effort is required to improve the biodegradability of PLA-containing systems, after the material has fulfilled its specific role in the application.

Polymer composites processes, in turn, are multiphase systems formed by two or more components, generally polymeric and one non-polymeric [104,108]. When at least one of the phases of this composite has nanometric dimensions, it is called a nanocomposite [104]. Unlike blends, the constituents of a composite interact with each other through strong chemical and physical reactions [108]. A literature review by Murariu and Dubois highlights recent and current developments, results, and trends in the field of PLA-based composites [109]. In addition, a comprehensive review of PLA composites reinforced with synthetic and natural fibers has been published by Ashothaman et al. [110]. In this review, the authors mainly address some methods of manufacturing polymeric composites (especially using compression molding and injection molding methods) and reinforcement of PLA composites with different fibers, between natural fibers (treated or not) and synthetic fibers. They pointed out that PLA composites reinforced with synthetic fibers are more easily manufactured, since both are hydrophobic and have good compatibility, while natural fibers, due to their hydrophilic nature, have low adhesion or incompatibility with the polymer. PLA composites reinforced with bioactive glass fibers and magnesium, PLA biocomposites with cellulosic fibers treated by microwave and enzymatic treatment, PLA-based biocomposite reinforced with flex fiber with treated surface, PLA reinforced with maple wood flour, PLA reinforced with hydroxyapatite, among others were mentioned. The authors concluded, based on the cited studies, that it is possible to improve the mechanical strength, stiffness and crystalline behavior of PLA composites reinforced with these fibers.

## 5. Materials of PLA Produced from D,L-Lactic Acid and Their Applications

There are many articles in the literature dealing with materials involving PLA prepared by either D,L-lactic (D,L-LA) acid or rac-lactide (rac-LA). These materials have been published with a variety of objectives and applications. Thus, we will present some of the main achievements obtained in a sequence of the type of material, taking into consideration the use of either commercial PDLLA or synthetic procedures by the own authors.

### 5.1. Synthesis of PDLLA Using Different Catalysts

Xu et al., described the synthesis of PDLLA by polymerization of either D,L-lactic acid polycondensation or D,L-lactide ring-opening polymerization (ROP) using SnCl_2_ catalyst to produce low or high molar mass polymers, respectively [111]. It is known that Poly(α-hydroxycarboxylic acids), especially PLA and PGA (poly(glyco1ic acid)) together with their copolymers are the most known synthetic polymers used for biodegradable biomaterials. The production of low number-average molecular weights (M_n_) PDLLA showed an increase with time up to 20 h; after that, negligible effect was observed. Different M_n_ was obtained (0.5–2.3 kDa) by polymerization temperature control (up to 200 °C). Using ROP, a higher M_n_ was obtained (20–50 kDa) depending on the initial molar ratio of the dimer: initiator (D/I), time and temperature. At low initiator concentrations (D/I > 15,000), the M_n_ increased, and molar mass distribution became wider. As it is known in the literature, fine management of those parameters is essential to producing PDLLA.

Gao et al., reported the stereoselective synthesis of PDLLA, with predominantly isotactic chains, via direct synthesis by melt polycondensation of D,L-lactic acid in the absence of solvent [112]. The authors used a catalytic system consisting of Sn(II)/TSA (p-toluenesulfonic acid monohydrate) and obtained a polymer with the following characteristics: M_n_ of approximately 18 kDa, as determined by Gel Permeation Chromatography (GPC); 14% crystallinity; T_g_ of 48.4 °C and T_m_ of 104.8 °C, according to Differential Scanning Calorimetry (DSC) results, and degree of racemization of 40%, calculated from ^13^C NMR. Furthermore, polarimetry results corroborated by ^1^H and ^13^C NMR, XRD and DSC data indicated that isotactic chains with L-lactic acid units were predominant in this polymer, which is different from the results commonly reported for PDLLA synthesized by ROP, i.e., it is usually reported that amorphous polymer with random chains is formed [112].

Chafran et al., also performed the stereoselective synthesis of PLA using 12-tungstophosphoric acid (H_3_PW) supported on activated carbon (C) as the catalyst in the direct polycondensation of D,L-lactic acid (D,L-LA) [113]. After a series of tests, the optimal reaction conditions were determined (15 h without any extra solvent; 0.1 wt.% of the supported catalyst; 20% H_3_PW/C calcined at 400 °C). It was possible to obtain a polymer with M_w_ of 17.4 kDa containing up to 85% of L-lactic acid units (according to polarimetry, and also supported by XRD, ^1^H NMR, ^13^C NMR and DSC data), demonstrating the stereoselective character of the synthesis. The polymer obtained showed a double melting peak, which was justified as a result of the formation of blends of PLLA and PDLA.

After the promising results obtained using the H_3_PW/C catalyst, Chafran et al., tested other porous matrices as support for the H_3_PW, such as silica (SiO_2_) and alumina (Al_2_O_3_) for the same melt polycondensation reaction of D,L-LA without using any solvent [114]. All three tested catalysts (H_3_PW/C, H_3_PW/SiO_2_ and H_3_PW/Al_2_O_3_) resulted in a crystalline PLA, as attested by several characterization techniques (e.g., XRD, DSC, ^1^H and ^13^C NMR). Confirmed by polarimetry, the synthesized PLA showed an enantiomeric excess of up to 95% of L-lactic acid units with 14.9 kDa of M_w_ (obtained by GPC). These results were attributed to the catalytic activity conferred by the different acid sites (Brønsted and Lewis acid sites, strength, and distribution) on the surface of the catalysts. The presence of H_3_PW is important, since without this heteropolyacid, the supports of silica, alumina and activated carbon only produce OLA (oligomers of lactic acid) with M_w_ below 9 kDa. An inversely proportional relationship was observed between the strength (based on enthalpy measurements) of the acid sites of the catalysts and M_w_ of the polymers, with the milder acid catalyst (H_3_PW/C) being the one that led to the PLA with the highest M_w_, followed by H_3_PW/SiO_2_ and H_3_PW/Al_2_O_3_. It is believed that despite the enantiomeric excess presented, the polymers obtained consisted of blends of PLLA and PDLA formed due to the binding of blocks (oligomers D and L) on different acid sites on the surface of the catalysts. This was evidenced mainly by a double melting peak (T_m_) attributed to the crystalline domains of PLLA and PDLA [114].

**Figure 3:**
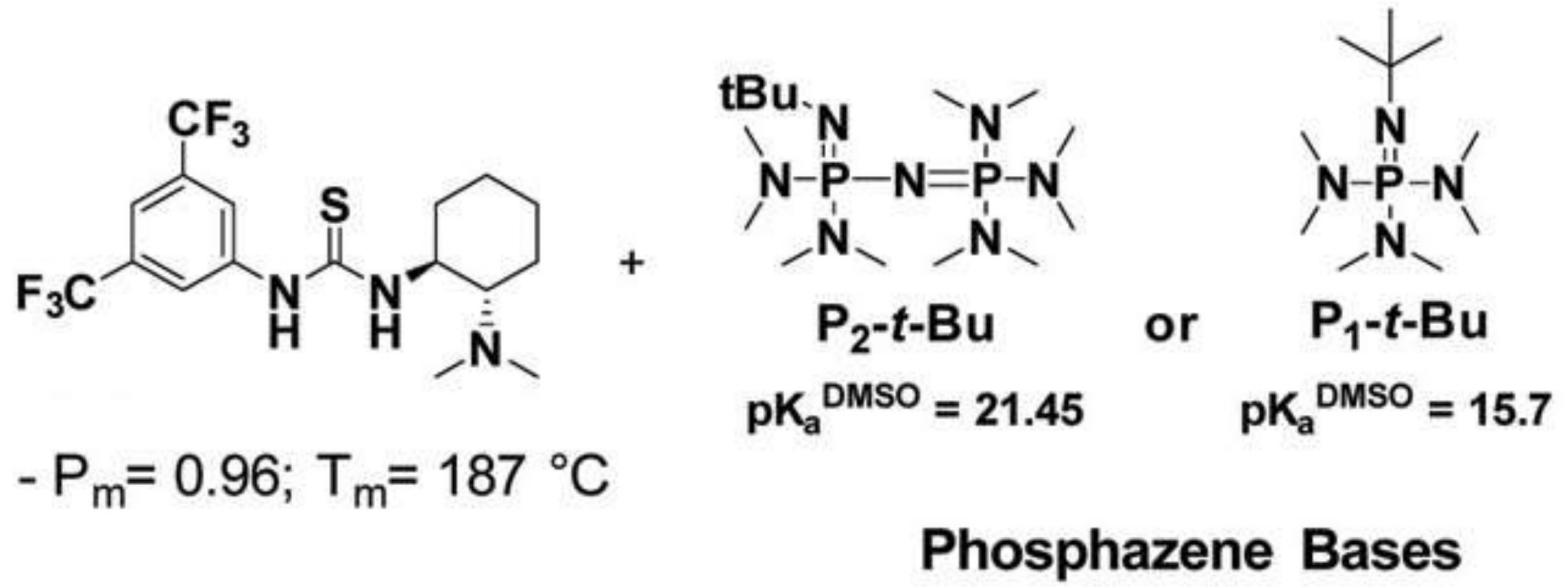
Organocatalysts for stereoselective ROP of rac-LA, adapted from reference [115]. Copyright @ 1996 RSC, License Number: 1220244-1.

Zack et al., used a chiral catalyst to produce PLA from rac-LA at room temperature [115]. They use the chiral Takemoto’s catalyst [116] in conjunction with phosphazene bases to form a unique chiral binary organocatalyst system to form PLA (Figure 3). This combination enabled remarkably high organocatalytic activity and stereoselectivity for the ROP of rac-LA, yielding metal-free PLA with melting temperature as high as 187 °C, and a probability for forming meso dyads (Pm) of 0.96. Some organic bases were paired with Takemoto’s chiral thiourea-based organocatalysts (TUCs). ROP reactions were carried out in the following addition order: phosphazene base and the TUC, followed by benzyl alcohol (BnOH) and rac-LA at room temperature. It used dichloromethane (DCM), tetrahydrofuran (THF) or toluene as solvent. First, the reaction was investigated in toluene using P_2_-t-Bu and P_1_-t-Bu alone ([rac-LA]^0^/[BnOH]^0^/[Base]^0^ = 200/1/1), where P_i_ represents different catalysts. P_2_-t-Bu and P_1_-t-Bu achieved 90% monomer conversion in 30 and 120 min, respectively, forming PLA narrow dispersions (1.2 and 1.3, respectively). The PLA synthesized from phosphazene bases used alone were found to be atactic, indicating the absence of stereocontrol at room temperature during the reaction. The ratio TUCs/bases were increased from 1/1 to 4/1 (relative to the phosphazene) aiming to optimize catalytic performance and stereoselectivity gain. Using P_2_-t-Bu paired with TUCs, the ROP of rac-LA in toluene proceeded under homogeneous conditions at 25 °C and monomer conversions reached 90% within 4 h. The polymerizations that were carried out in THF or DCM produced atactic PLA, under otherwise identical conditions to those used in toluene. Stereocontrol was achieved by the enantiomorphic site control (ESC) mechanism, providing metal-free and highly crystalline PLA in the 25–80 °C temperature range.

**Figure 4:**
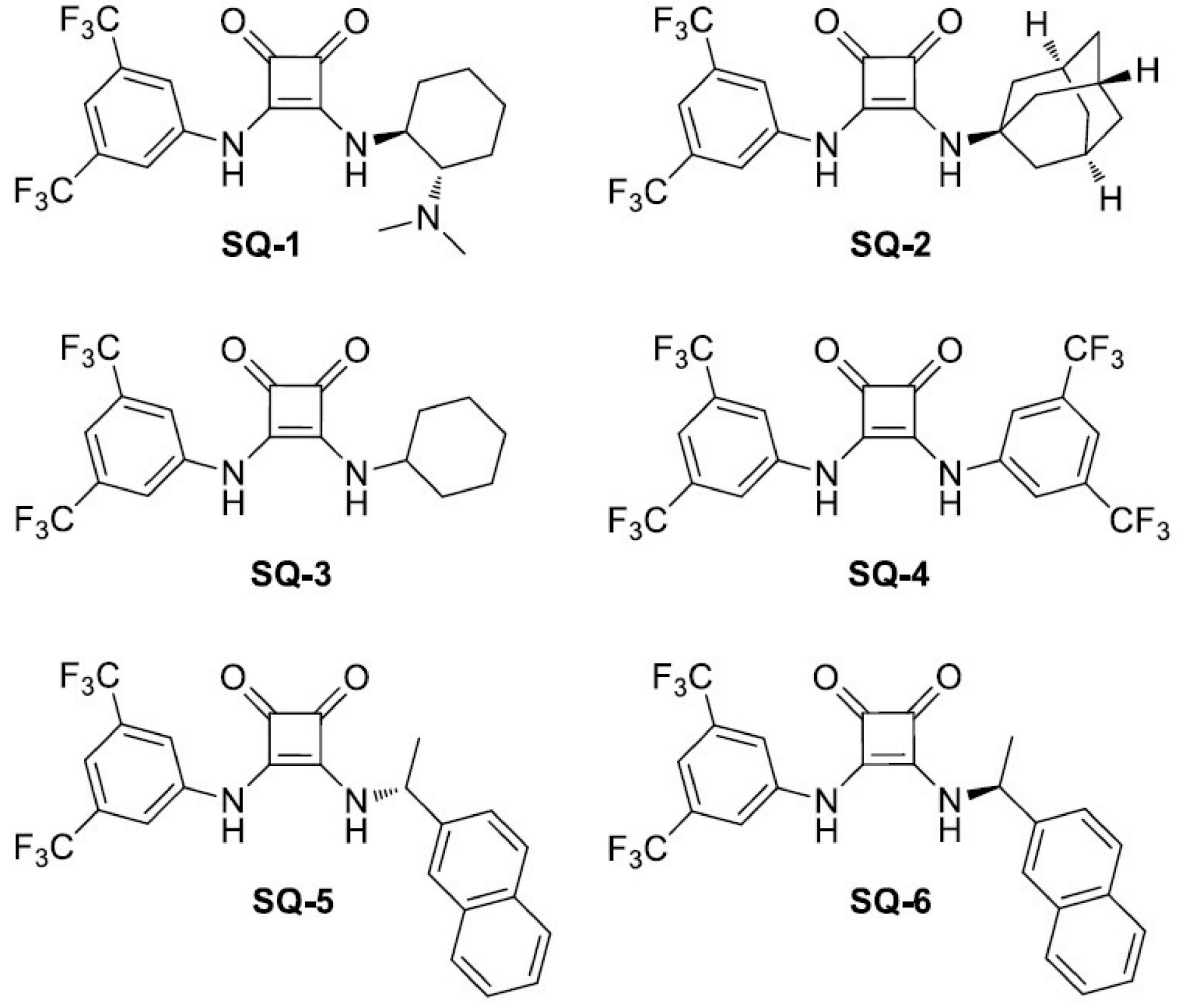
Structures of squaramide-based organocatalysts employed in the work of Jiang et al. [117] for stereoselective ROP of rac-LA. Copyright @ 2022 John Wiley and Sons, License Number: 5303791423435.

Jiang et al., prepared stereoselective ROP of rac-LA at room temperature using squaramide-based catalysts, which are part of the efforts toward the development of metal-free catalysts [117] for this reaction. The authors explored the catalytic properties of a series squaramide-based catalysts containing chiral or bulky substitutes, respectively (Figure 4). The structure SQ-1 was inspired by the thiourea-amine catalyst and its activity was analyzed in different solvents, being rather low in THF and toluene but much faster in dichloromethane (DCM), with 51% monomer conversion in 24 h to generate PLA (rac-LA/SQ-1/BnOH = 100/5/1, M_n_ (obtained by GPC) = 5.9 kDa; dispersity (D) = 1.08, and parameter by enantiomorphic site control method (ESC), P_i_ = 0.88). Probably, DCM had better solubility towards catalyst and PLA at room temperature. PLA polymer showed a melting temperature of 153 °C, indicating its semi-crystalline character. After polymerizing for 72 h under the same condition, the monomer conversion was increased to 95% and the isotacticity of the resulting PLA remained at the same level, i.e., M_n_ (GPC) = 11.5 kDa, D = 1.38, P_i_ (ESC) = 0.85. When the SQ-1 increased from 5 to 10 mol%, monomer conversion increased to 88% in 24 h, but the stereoselectivity of the polymerization did not drop when it happened (M_n_, GPC) = 12.1 kDa, D = 1.08, P_i_ (ESC) = 0.85. The bifunctional catalyst SQ-1 presented the ROP of rac-LA with high controllability but low activity. Thus, the authors used a binary catalytic system containing squaramide and a stronger organic base to make the polymerization more efficient (SQ-2 to SQ-6 and 1,8-diazabicyclo[5.4.0]undec-7-ene (DBU)). This binary system promoted the polymerization reaction at room temperature within a short reaction time, among which SQ-2 exhibited the best stereoselectivity without transesterification side reactions during the process.

Zhao et al., directly synthesized PDLLA starting from D,L-lactic acid and SnCl_2_ as the catalyst by melt polycondensation [118]. The authors used conditions such as 0.5 wt.% SnCl_2_, 170–180 °C, 70 Pa, 10 h of reaction, and obtained a viscosity-average molecular weight (M_n_) = 4.1 kDa. The D,L-LA was dehydrated for 6 h at 140 °C and 4000 Pa in a three-neck flask equipped with a thermometer and mechanical stirrer. After this, a catalyst was added, and the melt polymerization was carried out under the temperature range from 160 to 200 °C at 70 Pa for 5–20 h. In the end, purification after vacuum drying yielded a withe powder product (PDLLA). The produced polymer was used as microspheres for drug delivery material for erythromycin (ERY) and ciprofloxacin (CIP). The authors chose the PDLLA because it is amorphous and there is no residual microcrystallinity after degradation in vivo, which allows its use as a drug delivery material. ERY or CIP and PDLLA were dissolved in an organic solvent, after which the solution was placed into the dispersing medium. When the suspended mixture system was uniform through stirring, the diffusing agent was added to form microspheres (MS). ERY-PDLLA-MS and CIP-PDLLA-MS were prepared by collecting the microspheres through filtration and washing them with distilled water after being dried in a vacuum. The authors found that 0.5 wt.% SnCl_2_ as a catalyst, 70 Pa, temperature 170–180 °C, and 10 h produced the highest M_n_ of PDLLA. Furthermore, ERY–PDLLA–MS had a good spherical shape, with a half-time (*T*_1/2_) of 51 h. After 175 h, the accumulated release percentage was 80.0%. The test in vivo showed that ERY–PDLLA–MS was more easily distributed in rabbit lung tissue. The release time (T_1/2_) in vitro of CIP–PDLLA–MS was 24.9 h. After 53.2 h, the accumulated release was 84.0%. The system in vitro of CIP–PDLLA–MS showed a significant sustained release.

Hosseyni et al., synthesized PDLLA from the melt polycondensation of D,L-lactic acid and its surface was modified with triazine dendrimers in a one-step process [119]. In order to obtain a polymer with high molar mass, the oligo lactic acid (OLA) was synthesized and the melt polycondensation of OLA occurred in the presence of three different catalytic systems: tin(II) chloride dihydrate and p-toluenesulfonic acid (TSA); tin(II) chloride dihydrate and boric acid mixture, and antimony(III) trioxide. This modification enhances the ratio of surface area to volume, as well as increases the number of functional groups. The system of tin(II) chloride dihydrate and TSA produced the polymer with higher molar mass compared to the other catalysts (39.6 kDa), reaction time of 8 h, T_m_ of 150 °C, and T_g_ of 61 °C, which was attributed to the high solubility and activity of this catalyst in the presence of the melt lactic acid. The ^1^H NMR spectra of the grafted PDLLA with each of the dendrimers was demonstrated by the PEG-related signals, PDLLA, and triazine proton signals. The hydrophilic character of PEG and the presence of grafted dendrimers increased this character on the PDLLA, which makes them potentially biocompatible with enhanced hydrolytic degradation capability.

Gierej et al., used PDLLA (PURASORB PDL 20, from Corbion Purac Biomaterials) to produce flat PDLLA plates using a compression molding device under a vacuum [120]. They make samples with uniform thickness and low surface roughness, in order to obtain transparent polymer plates with a diameter of 15 mm and thickness of 0.65 to 1.00 mm, using 0.3 to 0.6 g of granulates. The authors also fabricate fiber preforms using a melt-casting process in dedicated Teflon molds with granulated PDLLA and placed in a vacuum oven at 180 °C for several hours, until the material was fully melted, leading to the appearance of vacuum voids because of the shrinkage of the material upon cooling. After this, they produced the PDLLA fibers with a standard heat drawing procedure and fully characterized the materials by measuring their optical transmission and specular reflectance spectra. The authors achieved PDLLA fibers with an attenuation of 0.11 dB/cm at 772 nm and that the thermo-optic coefficient is in the range of −10^−4^/°C. Accordingly, they claim that this value is lower than others reported at that time for other polymer biocompatible waveguides. They also studied the degradation of PDLLA fibers in vitro, revealing that the one with the largest diameter (600 μm) degrades faster than those with smaller diameters (300 and 200 μm) and shows more than 84% molecular mass loss over a period of 3 months. These characteristics make it possible to use this material to deliver light in vivo for periods of several hours and that can be left inside the body to degrade for up to two to three months. Light-based in vivo therapies currently require invasive treatments, but the use of biodegradable optical fibers would enable much less invasive therapies, according to the authors.

Wu et al., investigated the optimal thermal parameters of the fused deposition modeling (FDM), which is a commercial 3D printing process, for microfabrication of PLA microneedles (MN) [121]. MN is a recent medical device used to pierce the stratum corneum, targeting the epidermis and dermis layers of the skin. Conventional MN structures need a post-fabrication process to reduce the size of PLA needles printed via FDM, and hydrolysis in an alkaline solution is a feasible approach for this. Moreover, weak bonding between PLA layers during additive manufacturing triggers the detachment of PLA needles before etching to the expected sizes. The authors used a commercial PLA filament (16609, Mutoh, Japan) with defined thermal properties (density of 1240 kg/m^3^, T_g_ of 60 °C, T_m_ of 170 °C, and decomposition temperature (T_d_) of 220 °C). The authors also discussed that commercially available PLA for practical FDM processing comprises a mixture of L- and D-lactic acids (PDLLA) but with a major proportion of L-lactic acid, probably due the PLA desired to be strictly amorphous and with a molar mass of 50–140 kDa. These properties are required to stabilize the viscosity of molten PLA during the extrusion process.

Wang et al., reported a new method to create biodegradable and reusable fibrous mask filters made by a true nanoscale PLA fiber (average size of 37 ± 4 nm) fabricated via electrospinning of a dilute solution [122]. Because of the occurrence of the coronavirus pandemic, the use of individual face masks with efficient performance has increased. In this sense, precursor solutions were prepared using PLA (M_r_ = 200,000, from NatureWorks, Co., Blair, NE, USA), tetrabutylammonium chloride (TBAC), dimethyl carbonate (DMC), and dimethylformamide (DMF). To fabricate scaffold nanofibers, different quantities of TBAC were dissolved in DMC/DMF and stirred. Subsequently, PLA was added to the TBAC solution at different concentrations and stirred. After this, the electrospinning process was conducted using a modular electrospinning machine. The resultant mask filter exhibited a high filtration efficiency (PM_0.3_-99.996%), which is more breathable (air resistance of 104 Pa). In addition, after exposure to an outdoor humid environment for 100 h, it had a robust filtration efficiency (humidity 90%, filtration efficiency > 99.99%) that was much higher than that of commercial 3M (N95) masks. Moreover, in contrast to commercial masks, the resulting filter and the multiscale structured nanofiber membranes completely degrade after 150 days of outdoor soil burial.

### 5.2. Synthesis of Blends, Copolymers and Composites Using PDLLA

Chaubey et al., synthesized, characterized and studied the antimicrobial activity of lignin-PLA blended film [123]. The PLA was synthesized by the ROP method of commercial aqueous lactic acid using stannous chloride as the catalyst. The blended film was prepared with a mixture of 1% poly(vinyl alcohol) (PVA), 0.5% lignin-PLA blend, and 1% poly(ethylene glycol) (PEG). The reaction mixture was cast onto a plastic tray and left for 3 days at room temperature to dry out. Finally, the uniformly dried films were used for structural and functional characterization (film thickness, moisture content, water solubility, swelling ratio, transparency, mechanical properties, and soil degradation). The antimicrobial activity was probed by the agar diffusion method using gram-positive bacteria (Staphylococcus aureus, Bacillus circulans, Staphylococcus oralis and Ralstonia eutropha). DSC study indicated that the addition of lignin shifted the glass transition temperature of neat PLA (T_g_ = 98.29 °C) to 133.13 °C, followed by an endothermic event with ΔH value of 475.75 J/g to 271.52 J/g, respectively. The TGA and DTG scans identified five different steps to PLA film: first, from 59 to 100 °C, due to the moisture; second, between 174.2 °C (onset) and 225.6 °C (endset), due to the degradation of some low molecular mass molecules. Further steps showed the presence of high molecular mass molecules at higher temperatures ranging from 400 to 528 °C, whereas the lignin-PLA film showed the initial onset temperature and final degradation temperature from 67.8 to 476 °C. This variation indicated the lower thermal stability of lignin-PLA as compared to pure PLA. XRD patterns showed a broad diffraction peak for neat PLA at 2θ = 16.7°, which was shifted to 2θ = 15.3° and around 19° after lignin addition, and also with a decrease in the relative intensity as compared to pure PLA. This indicated that the amorphous phase grew in blend films due to lignin addition because it is itself an amorphous compound. Finally, a significant improvement in antimicrobial activity and biodegradability of PLA film was observed after the addition of lignin.

Ciarfaglia et al., have studied electrospun scaffolds, which are important materials for a variety of applications, including biomedical tissue engineering [124]. PLA is considered suitable for these applications, but many properties should be improved in order to be more effective. In this sense, scaffolds composed of commercial PDLLA (EasyFil PolyLactic Acid, transparent pellets, molecular weight 126,000 g/mol, density 1240 Kg/m^3^, from Form Futura, Amsterdam, The Netherlands), gelatin (GE), cellulose nanocrystals (CNCs) and an elastin peptide (El) were prepared by electrospinning using a variety of mixtures: GE and PDLLA (ratio 1:3, 12.0% wt./vol); CNCs, GE and PDLLA (with CNCs at 1, 3, 5 and 8 wt.% compared to the other components) and El, 8% CNCs, GE and PDLLA (EX15:CNCs:GE:PDLLA ratio 1:7.5:22.5:67.5, 13.1% wt./vol). Furthermore, electrospun scaffolds were cross-linked using EDC/NHS (N-hydroxysuccinimide/N-(3-dimethylaminopropyl)-N′-ethyl-carbodiimide hydrochloride, ratio 1:1) in an 85.5% ethanolic solution. All the components presented good miscibility. The addition of CNCs and El to the mixtures changed the concentration blends without affecting their properties and the electrospinning process. Moreover, the addition of CNCs up to 3 wt.% and El increased degradation temperature and hydrophilic properties. Finally, adding CNCs provided an increase in the glass transition temperature (T_g_) for the scaffolds series. A correlation between water melting temperature and average diameter of fibers was found after the swelling test. Scanning Electronic Microscopy (SEM) analysis verified that all the polymeric blends showed a similar morphology and an analogous behavior after cross-linking reaction, but the crystalline phase (T_m_ values) was less affected than the amorphous phase (T_g_ values).

**Figure 5:**
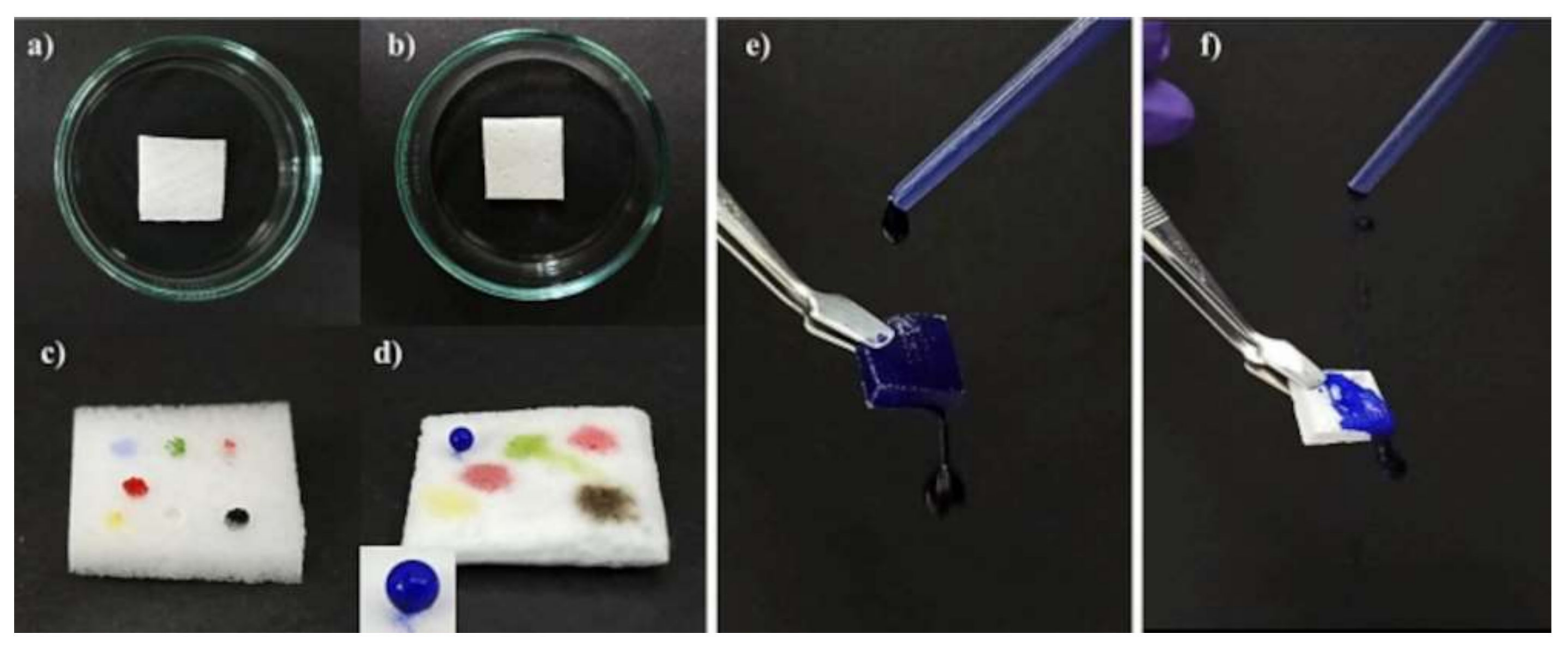
(**a**) Pristine melamine (PM), (**b**) PCL/PDLLA (80:20) melamine sorbent, oil and water absorption of (**c**) pristine melamine and (**d**) PCL/PDLLA (80:20) (bluewater, red-hexane, maroon-octane, dark yellow-soybean oil, light yellow-motor oil, green-petroleum ether, and black crude oil). Water absorption of (**e**) pristine melamine and (**f**) PCL/PDLLA (80:20). Obtained from reference [125]. Copyright @ 2021 Elsevier, License Number: 5303800945222.

Arunagiri et al., investigated the effects of an eco-friendly blend of polycaprolactone/Poly-D,L-lactic acid (PCL/PDLLA) modified with melamine sorbent on oil absorption and emulsified oil-water separation [125]. This potential application for the material was raised because accidental crude oil spillages have a huge impact on the living organisms in ocean waters. In this sense, PCL was blended with PDLLA in different mass ratios (90:10, 85:15, 80:20) to obtain potent hydrophobic polymer solutions. The chemical and physical stability of the polymer-coated membrane sample were tested in harsh environments. The authors also performed oil absorption experiments using melamine as a substrate and dipped it to achieve interconnected porous morphology by a facile freeze-drying technique. They verified that PCL/PDLLA modified melamine sorbent exhibited oil absorption capacity ranges of 3.3–8.7 g/g to distinct viscous oils and oil purity of over 99% along with an emulsified oil separation. PCL/PDLLA (80:20) exhibited significant oil absorption and emulsified oil rejection properties, with enough physicochemical stability and reusability (Figure 5).

Tien et al., used X-ray computerized tomography (CT) to analyze the three-dimensional structure of huge spherulites of pure poly(oxyethylene) (PEG) and blends with racemic random copolymer amorphous PDLLA (20 and 50 wt.%, referred to as DL20 and DL50, respectively) [126]. Spherulite consists of needle-like crystals that are frequently found in inorganic materials and small organic molecules. They are interesting materials for properties related to direction and symmetry. Pure PEG sample and its blends with PDLLA (Sigma-Aldrich, Burlington, MA, USA, Mw ~ 75,000–120,000 g/mol) were dissolved in DCM, yielding a solution with ~5 wt.% polymer concentration. The as-cast samples were prepared by solution casting for ~24 h at room temperature. The evaporation rate of solvent was suppressed by covering the casting Petri dish with aluminum foil with small holes. No large spherulites were observed in DL20 or DL50 samples probably because of the amount of amorphous PDLLA blended. It was possible to observe details of the spherulites in 3D by X-ray CT. 100% PEG spherulites were modeled as a curled bundle of lamellar crystallites.

Arenaza et al., have been working on Molecular Dynamics Simulations to predict the properties of polymer materials, especially PLA [127]. The combination of this method with Flory-Huggins theory allows the analysis of polymer blends. In this report, the authors described the results of the Simulation for blends of Poly(DL-Lactide) (PDLLA) with styrene-co-vinyl phenol copolymers (STVPh) blends of different compositions. Earliest, they confirmed the immiscibility of the PDLLA/PS blends, but later they found that PDLLA is completely miscible with poly(vinylphenol) (PVPh) due to attractive hydrogen bonding interactions between the hydroxyl groups (−OH) of PVPh and the carbonyls groups (−C=O) of PDLLA. The theoretical results have been compared with the available experimental information, and there was a good agreement between both data. This indicates that this theoretical method is a valuable tool for predicting the miscibility and phase behavior of polymer blends.

Pini et al., studied CO_2_ sorption and swelling isotherms that were measured at 35 °C and up to 200 bar pressures in a variety of PDLLA and poly(lactic acid-co-glycolic acid) (PLGA) polymers, which are attractive materials to make scaffolds [128]. Six different commercial polymers were used in this study: PLA15k (Resomer, Boehringer Ingelheim, Germany) and PLA52k (Purac, Amsterdam, The Netherlands); and four PLGA: PLGA8515 (85 mol% LA-15 mol% GA, glycolic acid); PLGA6535 (65 mol% LA-35 mol% GA) from Lakeshore Biomaterials, Birmingham, AL, USA; and PLGA7525 (75 mol% LA-25 mol% GA); PLGA5050 (50 mol% LA-50 mol% GA) from Resomer, Boehringer Ingelheim, Germany. The authors chose PDLLA and PLGA polymers because they can be finely tailored to control pore size and structure to host tissue formation from cell populations. The Sanchez and Lacombe (SL) equation of state was used to simulate the experimental swelling and sorption data. The authors did not observe a noticeable influence of the molar mass of the polymers on sorption and swelling and two homopolymers PLA15k and PLA52k showed fully equivalent behavior. Furthermore, PDLA polymers presented the strongest affinity for CO_2_ (sorption and fractional swelling) up to 0.55 and 0.68 g CO_2_/g polymer, respectively, at 200 bar pressure.

Li et al. [129] prepared composite scaffolds of commercial PDLLA (MW = 72 kDa, Institute of Medical Device, Beijing, China;) with bioactive wollastonite (prepared by chemical coprecipitation method) by the conventional solvent casting-particulate leaching method [130]. The authors verified that the scaffolds were bioactive and showed the ability to compensate for the pH decrease caused by the acidic degradation of PDLLA by-products. Furthermore, the hydrophilicity of the pure PDLLA was improved by adding wollastonite. The authors were able to prepare crystalline polymer foams and control porosity and surface/volume ratio with an open-cell morphology. They prepared membranes of porosity as high as 0.93, showing that this method was capable of the preparation of highly porous polymers.

**Figure 6:**
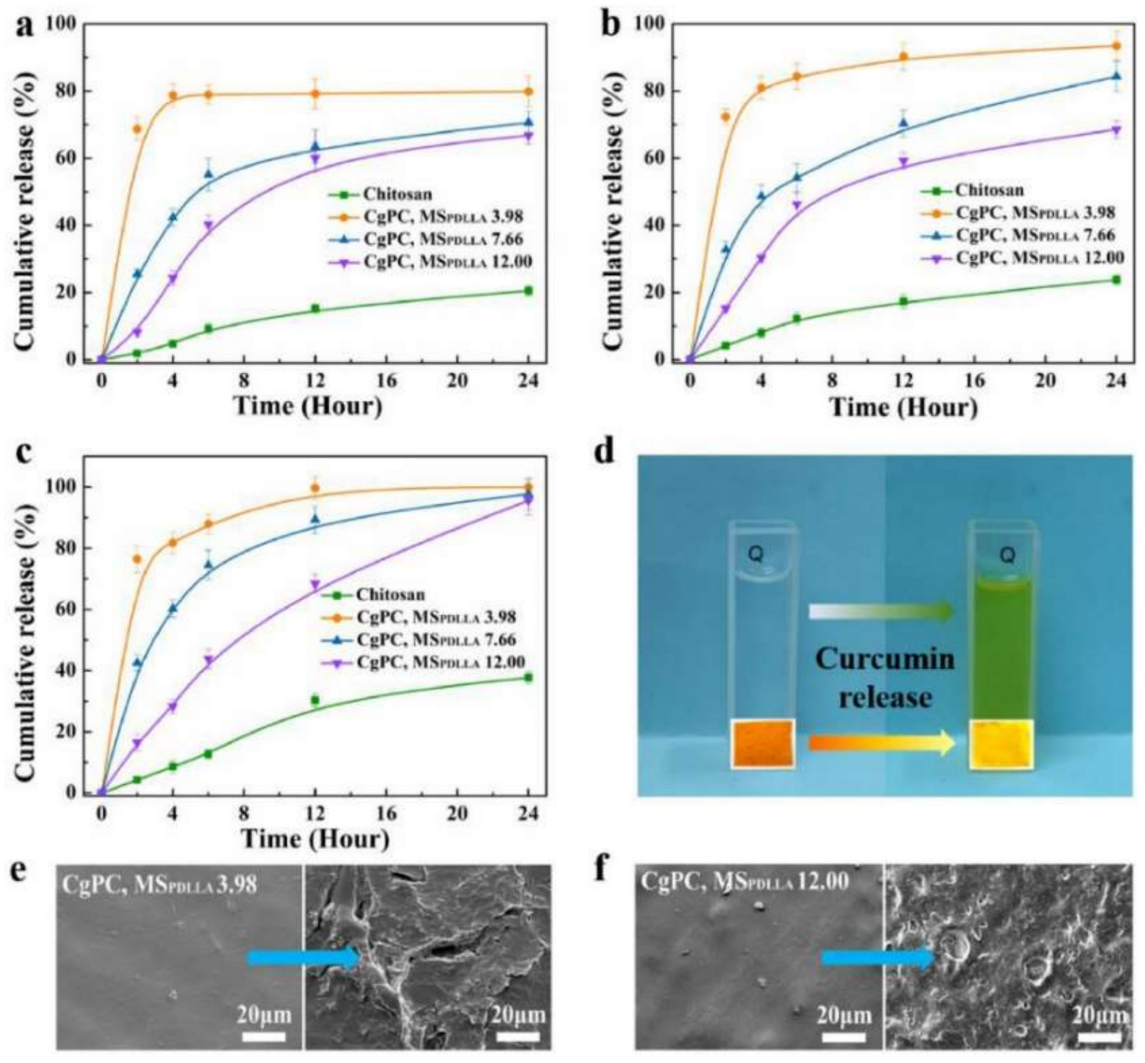
Drug release from chitosan and CgPC films in PBS (**a**), lysozyme (**b**) and porcine pancreas lipase (**c**). Color changes of the medium and film after curcumin release (**d**). SEM images of curcumin-loaded CgPC films (MS-PDLLA 3.98) before and after drug release in porcine pancreas lipase (**e**). SEM images of curcumin-loaded CgPC films (MS-PDLLA 12.00) before and after drug release in porcine pancreas lipase (**f**). Obtained from reference [131]. Copyright @ 2021 Elsevier, License Number: 5305970853110.

Sun et al., prepared chitosan-graft-PDLLA copolymers (CgPCs) successfully by ring-opening graft copolymerization of commercial D,L-lactide (98%, Aladdin-reagent Inc., Beijing, China) with chitosan [131]. They aimed to use a biodegradable polymer as a drug carrier, which is important in this application to a fine control of the degradation rate. For the synthesis, the authors used an eco-friendly system of 1-ethyl-3-methyimidazolium acetate/4-dimethylaminopyridine (EmimAc/DMAP). EminAc was chosen because of its ability to provide better chitosan solubility and maintain good fluidity even at a high feed ratio, whereas DMAP has a powerful catalytic ability in ROP. The degree of polymerization of D,L-lactic acid (DP-PDLLA), degree of substitution (DS-PDLLA) the molar substitution of PDLLA (MS-PDLLA) and the weight content of PDLLA(W-PDLLA) of CgPCs reached as high as 2.91, 4.13, 12.00 and 84% within 5 h, respectively. These copolymers showed an improved solubility in water, dimethyl sulfoxide (DMSO), DMF, Dimethylacetamide (DMA), methanol, ethanol, and methylene chloride due to the introduction of PDLLA, which destroyed the hydrogen bonds of chitosan. The drug release test from chitosan and CgPC films in PBS can be illustrated in Figure 6. The biodegradation rate of CgPCs was regulated by the MS-PDLLA, i.e., the value of 3.98 showed good hydrophilicity and fast degradation, whereas 12.00 exhibited relatively poor hydrophilicity and slower degradation. Hydrophobic curcumin was chosen as a model drug to investigate the drug release during degradation. Thus, the authors found that CgPCs had higher enzymatic degradability, particularly when exposed to porcine pancreas lipase, leading to a complete release of curcumin within 24 h. Moreover, these copolymers with different MS-PDLLA showed great variability in hydrophilicity and degradation rate, resulting in different drug release behavior.

Wang et al., synthesized poly(lactic acid)-poly(phenyl phosphate) via direct polycondensation starting from commercial D,L-lactic acid (Guangzhou Chemical Reagent Factory, Guangzhou, China), ethylene glycol (EG) and phenyl dichlorophosphate (PDP) [132]. PLA and poly(phosphate ester) have applications in biomedical fields and the authors aimed to combine the advantages of each material in the synthesis and potential use of copolymer poly(lactic acid)-poly(phosphate ester). This study compares and investigates two pre-polymerization methods. In the first, after LA and EG were uniformly mixed as preplanned feed molar ratio, the mixture was directly dehydrated for 6 h at 140 °C and 4000 Pa. Then, PDP was added and further dehydrated at 140 °C and 4000 Pa for 4 h. In the second pre-polymerization method, LA, EG and PDP were pre-polymerized together for 8 h at 140 °C and 4000 Pa. After pre-polymerization, the selected catalyst was added, and the melt copolymerization was carried out. In the solution of copolymerization reaction, when the first pre-polymerization was partially finished before the addition of PDP, the prepolymer was dissolved and its mixture with triethylamine (NEt_3_) was cooled with an ice bath, and then the solution of PDP was dropped inside. Finally, the purification and precipitation gave a white powder product after the sample has been vacuum dried. The catalyst choice in the direct melt polycondensation of LA is very important because it is inevitable to form HCl, so that NEt_3_ could be used to absorb it. Once the absorption product has a certain catalytic activity, the choice may be further improved. Metal oxides and chlorides, such as SnO, SnCl_2_, ZnO and ZnCl_2_ were also chosen as catalysts. However, the catalyst SnO could not give any copolymer and then ZnO was used in the experiments. The authors verified that when LA and EG were first dehydrated and then pre-polymerized with PDP, the direct melt polymerization obtained higher M_w_ than the direct solution polymerization method. When the feed molar ratio of LA/EG/PDP was 20/1/2, ZnO showed good catalytic activity and the direct melt copolymerization of LA, EG and PDP under 70 Pa and 160 °C for 8 h gave copolymer with the maximum M_w_ = 9.2 kDa. When the feed molar ratio of LA/EG/PDP was 20/1/1, for 8 h co-polycondensation under 160 °C and 70 Pa gave a copolymer with M_w_ = 27.7 kDa.

Wang et al., synthesized the biodegradable material poly(D,L-lactide-cholate) via direct co-polycondensation [133]. In pre-polymerization, commercial D,L-LA (Guangzhou Chemical Reagent Factory, Guangzhou, China) and cholic acid (CA) were uniformly mixed at a fixed molar feed ratio and the mixture was directly dehydrated at 140 °C under 4000 Pa pressure in a flask equipped with a mechanical stirrer and thermometer. After this process, the selected catalyst was added according to the weight percentage of the dehydrated reactants. The melt copolymerization was carried out in a range of temperatures (130–180 °C), 70 Pa, for 2–10 h. When the reaction finished, purification and subsequent precipitation ordinarily produced a white powder after vacuum drying. For the CA/D,L-LA molar feed ratio of 1/64, the optimal synthesis conditions were: 8 h of pre-polymerization, 0.3 wt.% SnO catalyst, and melt co-polycondensation for 8 h at 160 °C. This gives rise to a novel star-shaped PDLLA modified by CA with the maximum M_w_ = 5.6 kDa at a yield of 51.9%. Thus, this CA-modified PDLLA provides greater cell affinity and possible applications in drug-release microspheres and tissue engineering.

Xu et al., recently revised sirolimus release for coronary stent application using biodegradable polymers [134]. Drug-eluting stents (DESs) are usual for the treatment of coronary artery disease, and the use of biodegradable polymers is very attractive because it reduces the incidence of late thrombosis after stent implantation. The safety and efficacy of stent treatments can be improved by the drug-release behavior of DESs. Biodegradable polymers with different properties show various drug-release behaviors and have attracted research attention. Molar mass, composition, glass transition temperature, crystallinity, and the degradation rate are important properties of this application. In this sense, PDLLA is a prominent candidate to be used in DESs because of its controllable mechanical and chemical properties e.g., tensile strength of 27.6–50 MPa; elastic modulus of 1–3.4 GPa; T_g_ of 50–60 °C; T_d_ of 12–16 months. In addition, it is still difficult to establish a suitable balance between a long-lasting drug release, fast endothelialization, and suitable degradation for biodegradable DESs. The authors point out that PLA, PDLLA, and PLGA are the current materials for this application, which were prompted by some groups that obtained promising results involving PLA. Particularly, PDLLA has an amorphous structure, which results in satisfactory distribution of the drug in their structure for a long release.

Gomes et al., used commercial nanoparticles of poly(D,L-lactic-co-glycolic) acid (PLGA, 50:50%, Sigma-Aldrich, Burlington, MA, USA) to insert trans-[Ru(NO)Cl(cyclam)](PF_6_)_2_ (cyclam = 1,4,8,11-tetraazacyclotetradecane), and [Ru(NO)(Hedta)] (Hedta = ethylenediaminetetraacetic acid) by using the double emulsification process [135]. They aimed to study materials that could release nitric oxide (NO) because it plays key roles in different physiological processes and pathologies. The expectation was that lipophilic drug substances could be loaded in those nanoparticles of PLGA. The results show that the effective encapsulation of hydrophilic drugs turns them into a potential formulation as anticancer agents under irradiation.

Lunardi et al., prepared and characterized a biodegradable material based on cresyl violet (CV)-loaded on PLGA nano/microparticles (CV-NP and CV-MP) [136]. They used commercial PLGA (50:50, Mw = 17 kDa, from Sigma Chemical, Burlington, MA, USA). CV was entrapped in PLGA by emulsification with 2.0% poly(vinyl alcohol) (PVA) to prepare the nanoparticles, whereas microparticles were prepared by mixing CV and PLGA in a mixture of methanol: dichloromethane (1:9 mL) and later placed on an aqueous solution containing 2.0% (wt./vol.) of PVA. They aimed to use CV as a fluorescent tracer. In this sense, the authors obtained high yield and entrapment efficiency with high stability of the fluorophore on PLGA. Encapsulation of drugs in PLGA is a great alternative to avoid therapeutic agents that may produce distinct side effects. The main results demonstrate that smaller PLGA particles reach different organs (e.g., kidney, lung, heart, liver) making them a promising vehicle for targeted delivery. Thus, the developed polymer-based particles have the potential for diagnostic and therapeutic applications.

Xu et al., proposed a material based on thermosensitive poly (D,L-lactide)-poly(ethylene glycol)-poly(D,L-lactide) (PDLLA-PEG-PDLLA) hydrogel (PLEL) capable of holding Prussian blue nanoparticles (PBNPs) that can be used to efficiently scavenging reactive oxygen species [137]. They aimed to use the resultant PBNPs@PLEL novel material to wound dressing, which was able to improve diabetic wound healing. Diabetes mellitus (DM) is globally increasing with an estimated 19–34% of incidence DM patients that will develop diabetic foot ulcers at some point in life [138]. Thus, the developed material has plenty of applicability. PLEL hydrogel was prepared by mixing at room temperature PEG (heated at 100 °C and evacuated to eliminate water), D,L-lactide, and Sn(Oct)_2_ in a cooled flask. This mixture reacted for 12 h at 140 °C under an argon atmosphere. The resultant PDLLA-PEG-PDLLA block copolymer was dissolved in ethanol and reprecipitated with cooled n-pentane. Dissolving PDLLA-PEGPDLLA in PB microemulsion, the PBNPs@PLEL was obtained. Analysis by SEM images showed that both PLEL and PBNPs@PLEL prepared materials displayed a homogeneous and much interconnected network.

**Figure 7:**
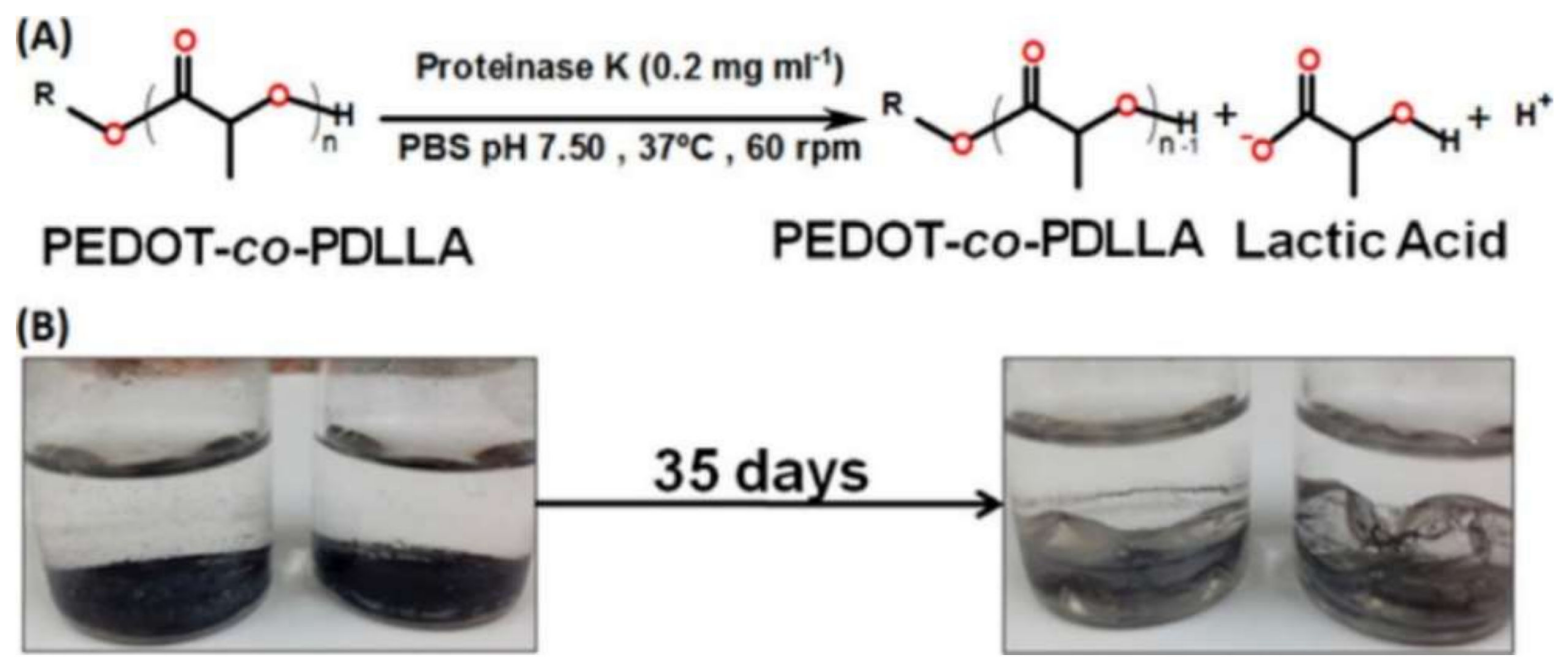
(**A**) Biodegradation Reaction and (**B**) Physical Aspects of PEDOT-co-PDLLA Films before and after 35 Days of Biodegradation Assay. Obtained from reference [139]. Copyright @ 2018, The Authors, ACS.

Da Silva et al., synthesized copolymers based on poly(3,4-ethylenedioxythiphene) with poly(D,L-lactic acid) (PEDOT-co-PDLLA) seeking novel electroactive biodegradable biomaterials [139]. The synthetic route of PEDOT-co-PDLLA involves an electroactive macromonomer of 3,4-ethylenedioxythiphene (EDOT), EDOT-PDLLA, using EDOT-OH as the initiator, and polymerization of PDLLA via ROP reaction catalyzed by Sn(oct)_2_. The reactions for PEDOT-co-PDLLA copolymers and images of the PEDOT-co-PDLLA films are shown in Figure 7. The results indicated that branched copolymer with conducting moieties was obtained. It was found that PEDOT-co-PDLLA materials had properties such as surface chemistry and charge density to make them potentially useful as scaffolds. In another application with PEDOT-PDLLA, Da Silva et al., also synthesized two inorganic/organic nanocomposites of Au [140]. The gold nanoparticles (7–8 nm of mean sizes), Au/PEDOT-PDLLA were able to the reduction of H_2_O_2_ in an aqueous solution with a limit of detection of 0.17 mmol/L, which makes it a promissory material for sensing.

Ayyoob et al., synthesized PLGA copolymers, called polyglactin, with three different chemical compositions (polyglactin 910, 815 and 820, with LA:GA ratios of 10:90; 15:85 and 20:80, respectively) by direct polycondensation of acid D,L-lactic and glycolic acid [141]. They used diphenyl ether as an azeotropic solvent and a bicatalytic system (SnCl_2_ 2H_2_O and methanesulfonic acid, MSA) under vacuum and obtained high molar mass PLGA, with corresponding viscosity values from 0.84 to 1.1 dL/g. The solubility of the copolymers increased with the content of D,L-lactic acid and decreased with the reaction time (possibly due to a higher molar mass and crystallinity of the copolymers). According to the authors, DSC results show that the percentage of crystallinity of the synthesized polyglactin 910 was 71%, which is higher than that presented by the polymer marketed by Ethicone, Vycril (which is a polyglactin 910). However, crystallinity decreased with increasing PDLLA content of polyglactin 815 and 820, reaching 63 and 17%, respectively. Furthermore, they reported that the T_m_ (obtained by DSC) increased with reaction time and decreased with increasing LA content.

Yang et al., prepared a bidentate dithiolpoly(ethylene glycol)-poly(D,L-lactide) (SH2-PEG-PDLLA) copolymer [142]. They aimed to use it in micelles that could be used in vivo as eye drops, in a way that this drug could more efficiently be absorbed by eye drop administration. The authors used dexamethasone as a model drug since this drug is usually applied to treat many eye conditions (e.g., conjunctivitis). The micelles containing a bidentate dithiol-terminated PEG corona were effectively prepared using newly synthesized 6,8-dimercaptooctanoate-poly(ethylene glycol)-b-poly(D,L-lactide). It was observed that dexamethasone was released and independent of the dithiol group with low cytotoxicity (below 10 mg/mL). Thus, the proposed formulation has the potential for topical ocular drug delivery.

**Figure 8:**
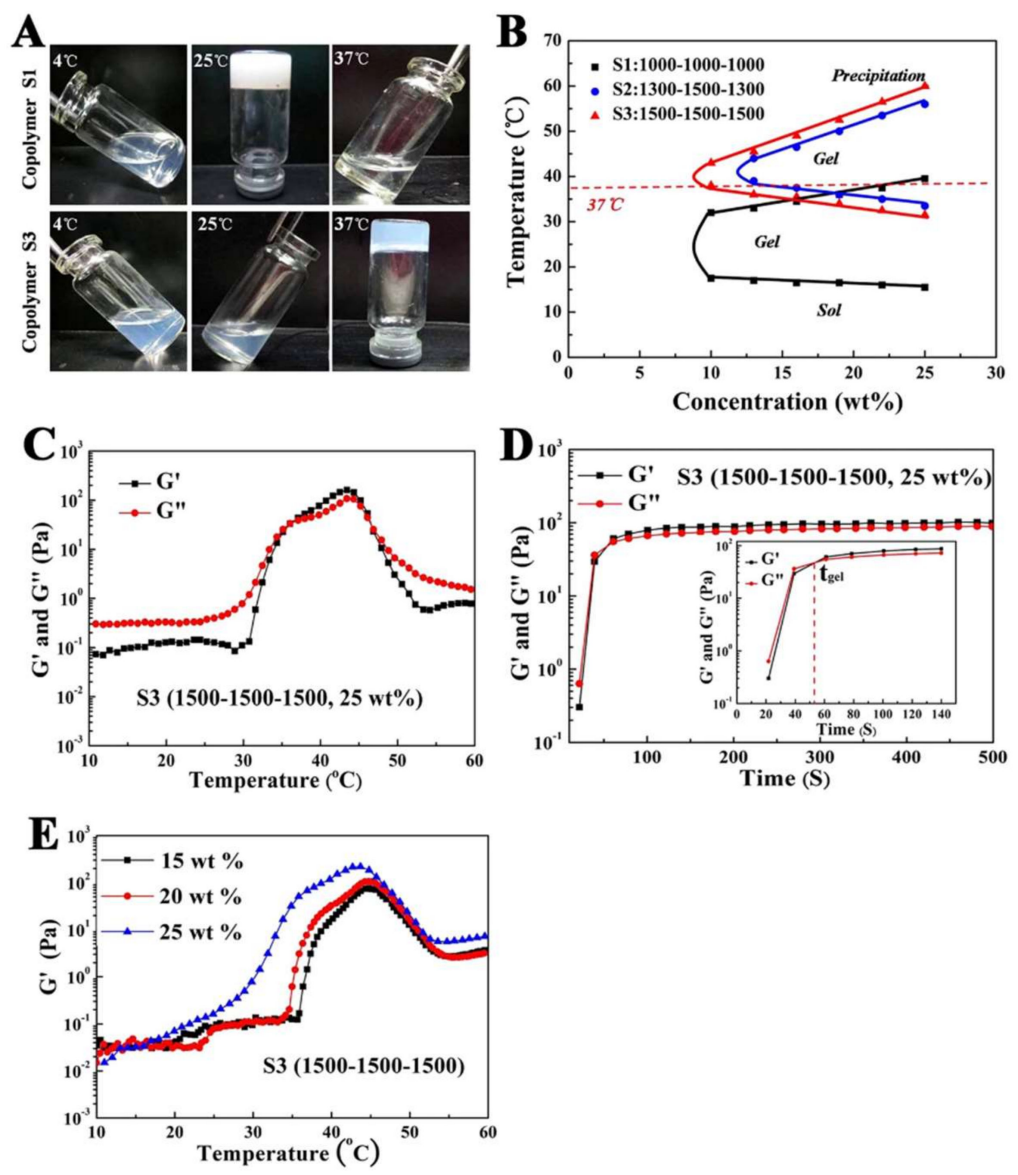
The thermogelling behavior assay and rheological analysis of the obtained. PDLLA-PEG-PDLLA hydrogel. (**A**) Photographs of the copolymer solutions at different temperatures. Copolymer L1000–E1000–L1000 (S1, 25 wt.%) gelates between 4 °C and room temperature and precipitated around 37 °C. Copolymer L1500–E1500–L1500 (S3, 25 wt.%) exhibited a sol at 4 °C and room temperature, and gelated around body temperature. (**B**) Sol–gel phase transition diagram of the hydrogel tested by the tube-inverting method. (**C**) Temperature dependence of storage (G’) and loss modulus (G”) for the copolymer aqueous solution (S3, 25 wt.%) as a function of temperature. (**D**) Gelation times of the copolymer solutions (S3, 25 wt.%) at 37 °C. (**E**) Change in G’ of the copolymer solutions at different concentrations (S3, 15 wt.%, 20 wt.%, 25 wt.%) as a function of temperature. Obtained from reference [143]. Copyright © 2022, The Authors, Springer Nature.

Shi et al., prepared a series of copolymers based on PDLLA-PEG-PDLLA in order to control the molar mass, block length and polymer concentration so that sol-gel transition behavior and the mechanical properties of the hydrogels could be fine-tuned [143]. This kind of temperature-sensitive polymeric hydrogels is claimed to be promising biomaterials for applications such as drug delivery, cell encapsulation, and postoperative adhesion prevention. The phase transition of the PDLLA-PEG-PDLLA copolymers aqueous solutions was investigated (Figure 8). The results indicated low cytotoxicity and hemolysis of this polymer, whereas the inflammatory response was adequate for small-animal. In vitro and in vivo degradation data demonstrated the integrity of the hydrogel for weeks.

Lee et al., synthesized 2-hydroxyethyl methacrylate (HEMA)-terminated PDLLA macromonomers (HEMA-PDLLA), which were further copolymerized with α-methylene-γ-butyrolactone (MBL) to produce their graft copolymers (PT-g-PDLLA) [144]. The goal was to examine their thermal properties and to develop the synthetic method of using macromonomers for graft and block copolymers. They were able to prepare the PT-g-PDLLA copolymers with various lengths and ratios, as well as HEMA-PDLLA having different lactyl chain lengths. Copolymers with two miscible states of PDLLA and PT and segregated domains were detected, depending on their M_n_.

Toshikj et al., proposed the synthesis of biodegradable triblock copolymers with PLA as the central building block, using three mechanisms (coordination-insertion, anionic and cationic) and also varying the nature of the catalysts (organometallic and organic) [145]. Two triblock polymers were prepared: poly(trimethylene carbonate) (PTMC): PTMC-b-PDLLA-b-PTMC; Poly(ε-caprolactone)s (PCL): PCL-b-PDLLA-b-PCL. The reaction system consisted of 100 mL-two-necked round bottom flasks equipped with a magnetic stirrer and a thermometer. They claimed that optimal conditions were obtained for the preparation of organized triblock copolymers starting from PDDLA as macroinitiators, without secondary reactions (e.g., transesterification). The mechanism of activated monomer was the most convenient methodology. The sequential copolymerization was obtained using consecutively 1,5,7-triazabicyclo [4.4.0] dec-5-ene (TBD) and methanesulfonic acid (MSA) catalysts, according to the PDLLA first route.

Chu et al., synthesized block copolymers based on monomethoxy-poly(ethylene glycol)-poly(ε-caprolactone-co-D,L-lactide) (MPEG-PCLA) with variable composition of poly(ε-caprolactone) (PCL) and poly(D,L-lactide) (PDLLA) [146]. The MPEG-PCLA were prepared by ROP of ε-CL and D,L-LA, using MPEG as an initiator and Sn(oct)_2_ as a catalyst. In addition, they tested the drug loading capacity of Docetaxel (DTX) on MPEG-PCLA micelles. The findings indicated that the higher content of the PDLLA block segment, the faster the hydrolytic degradation is, whereas acidic or basic solutions accelerate the degradation. Thus, the MPEG-PCLA copolymers can be designed with an adjustable ratio of PCL to PDLLA and can act as a potential drug delivery system.

**Figure 9:**
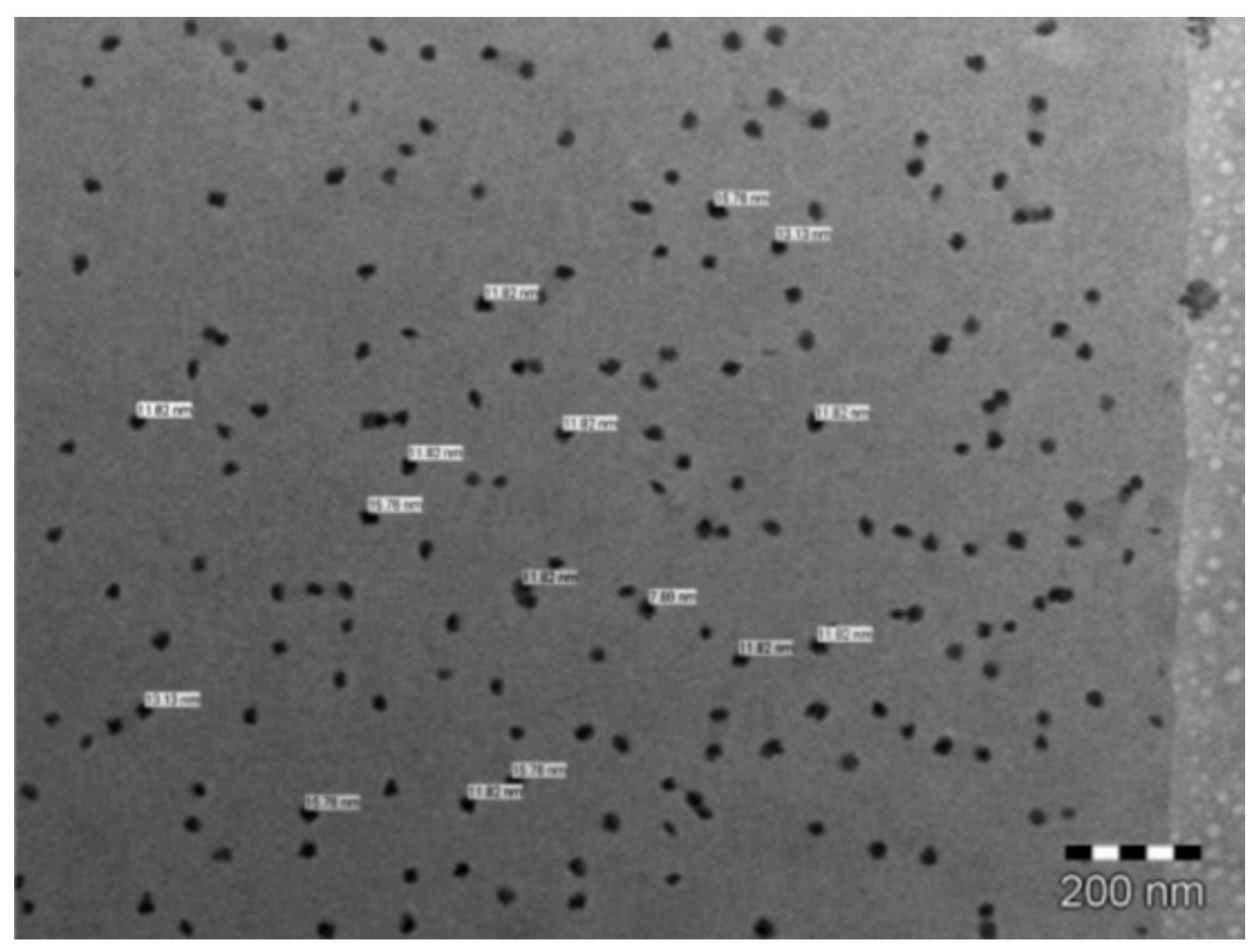
TEM image of the micelles obtained from aqueous solution of PDLLA42-b-PNVP63. Obtained from reference [147]. Copyright @ 2012 Elsevier, License Number: 5303821069340.

Ramesh et al., prepared amphiphilic poly(D,L-lactide)-b-poly(N-vinylpyrrolidone) (PDLLA-b-PNVP) block copolymers by ROP and xanthate-mediated reversible addition-fragmentation chain transfer (RAFT) polymerization [147]. The production of block copolymers is very important for applications such as drug delivery, nanoparticle preparation, coating, and compatibilizer agent in polymer blends. TEM images show the formation of micelle (Figure 9). The results reveal the presence of a single glass transition attributed to the miscibility of PNVP and PDLLA blocks, and that PDLLA was completely incorporated on the PNVP block copolymer. Thus, these data corroborate the successful formation of PDLLA-b-PNVP block copolymers.

**Figure 10:**
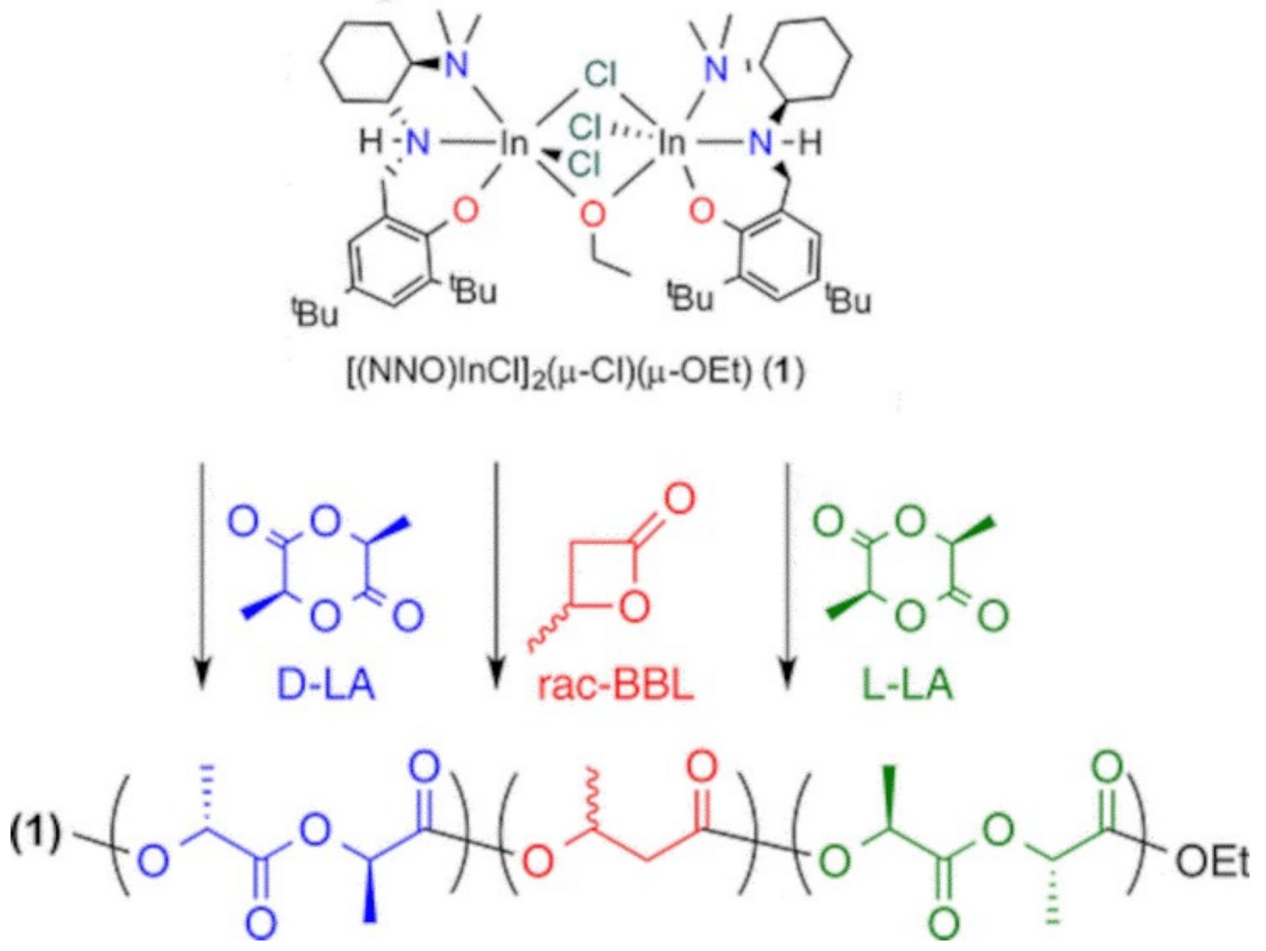
Formation of triblock PLA by sequential addition of LA. Obtained from reference [148]. Copyright @ 2013 ACS.

Aluthge et al., reported a sequential monomer addition method to prepare a series of triblock copolymers of PLA and poly(hydroxybutyrate) (PHB), and copolymers PLLA-PDLLA-PLLA and PLLA-PDLLA-PDLA [148]. The authors used ROP of cyclic esters lactide (LA) and β-butyrolactone (BBL) with a diaminophenoxy (NNO) supported dinuclear indium catalyst ([(NNO)InCl]2(μ-OEt)(μ-Cl)). The authors present the synthesis of nearly monodispersed diblock copolymers of DL-LA or D-LA with L-LA by ROP (Figure 10). The results indicated the stereocomplex formation of these copolymers with influence on their rheological and mechanical properties. The inclusion of PHB in the middle of the block provided more elastomeric nature (i.e., solid-like behavior), whereas the rheological properties of PLLA-PDLLA-PDLA and PLLA-PDLLA-PLLA blocks were comparable to those of pure PLLA, PLDA or their blends.

Sitompul et al., prepared poly(D,L-lactic acid)/poly(L-lactic acid) bentonite nanocomposites (PDLLA/PLLA-bentonite) [149]. PDLLA was synthesized using the direct polycondensation method under vacuum and ZnO as the catalyst. The authors obtained an average molecular weight of 92.2 kDa for the composite. The PDLLA/PLLA-bentonite nanocomposite films were synthesized by the solution-intercalation method using chloroform and sonication. The structure of the intercalated nanocomposite films was demonstrated by the XRD of the polymer films. Bentonite addition led to the improvement of mechanical properties, water vapor barrier and biodegradability. It was observed that different sonication times during the mixing processes of the polymer solution and the amount of bentonite affected the above-mentioned properties, i.e., mechanical, water vapor barrier, and degradation of PDLLA/PLLA-bentonite nanocomposite films.

**Figure 11:**
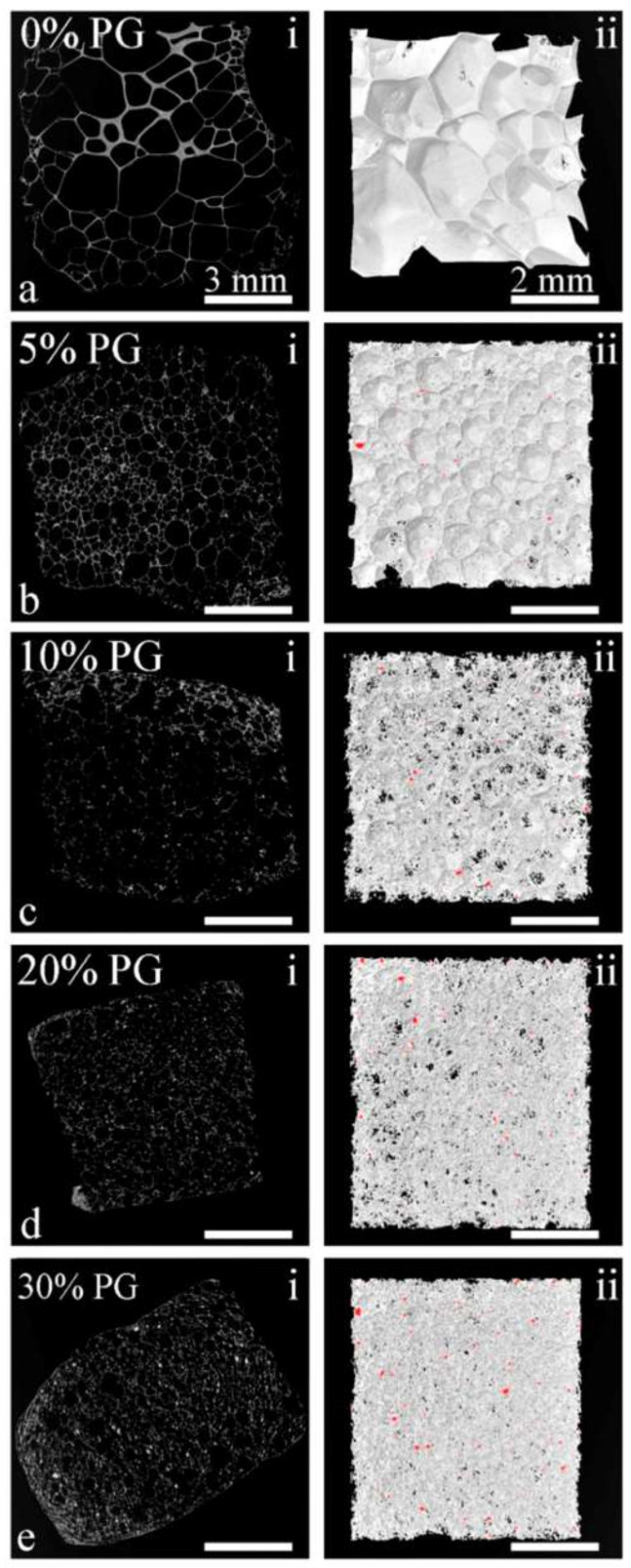
Micro-CT images of the SSF fabricated foams. (**a**) PDLLA, (**b**) PDLLA-5PGP, (**c**) PDLLA-10PGP, (**d**) PDLLA-20PGP, (**e**) PDLLA-30PGP. Note that, i and ii represent 2D and 3D images, respectively. The scale bars of the images on the left and right columns are 3 mm and 2 mm, respectively. Obtained from reference [150]. Copyright @ 2020, The Authors, MDPI.

Mohammadi et al., developed and used the solid-state foaming (SSF) method to sustainably produce highly porous PDLLA containing higher volume fractions of calcium phosphate-based glasses particulates (PGPs) [150]. In this method, the PGP filler was incorporated into commercial PDLLA (Purasorb^®^ PDL 05, Purac Biochem, Goerinchem, The Netherlands) at 5, 10, 20, and 30 vol.%. The authors aimed for a material that has appropriate mechanical properties and morphologies in terms of porosity (pore size, and interconnectivity) for potential bone tissue engineering (BTE) applications. Micro-CT 2D and 3D images of the SSF fabricated composite foams are shown in Figure 11. The method of dispersion revealed well-dispersed PGPs in the PDLLA matrix, according to SEM micrographs. PDLLA-PGP composite monoliths and foams with up to 30 vol.% PGP content were successfully created using the method of melt extrusion and compression molding followed by solid-state foaming working with CO_2_. This foaming process was revealed to be an efficient route to prepare PDLLA-PGP composites having macroporous structures (79 to 91% porosity). Thus, the produced materials are promising for bone tissue engineering applications.

Wang et al., developed a novel tumor-targeting Rapamycin (Rapa)-loaded membrane based on biodegradable PLA and polyethylene oxide (PEO) materials by electrospinning for the target treatment of malignant glioma [151]. Rapamycin is a mammalian target of rapamycin inhibitor and anti-proliferative agent. It is employed to treat glioma, one of the most common types of brain tumors in adults. It is a water-insoluble macrolide antibiotic administered to organ transplant patients to prevent rejection. The membrane materials with PDLLA (M_W_ = 200 kDa, Dai Gang Biological Engineering Co., Daigang, China) solutions with different loadings (5, 8,10, 12, and 15 wt.%) were prepared in polyethylene oxide (PEO) and a mixture of chloroform and DMF using electrospinning processes to produce the membrane. The drug-loaded process used the same method. FT-IR spectroscopy analysis demonstrated that Rapa was encapsulated in the polymer solution, and this process was highly efficient and stable over the range of drug concentrations. The in vitro drug release profiles and cytotoxicity were provided for its clinical applicability. However, additional research is necessary to confirm the mechanism of Rapa distribution within nanofibers, such as the effects of local rapamycin distribution.

Tudorachi et al., synthesized new nanocomposites of PLGA and magnetite (Fe_3_O_4_) [152]. PLGA was prepared by copolymerization and polycondensation of D,L-LA and GA (80:20 molar ratio) using [Sn(Oct)_2_] as the catalyst. The obtained PLGA/magnetite nanocomposite has a particle size of 864 nm and a saturation magnetization of 39.44 emu/g. This high value of magnetization suggests a possible utilization in the biomedical field. The polymer matrix PLGA can act as a shell to carry an active component, whereas Fe_3_O_4_ can be targeted using an external magnetic field. The prepared materials had good stability, based on the results of thermal degradation of PLGA using the TG/FT-IR/MS technique. Most of the degradation takes place in the range of 200–400 °C (mass losses higher than 90 wt.%), which was attributed to the cleavage of chemical bonds in the PLGA structure such as end OH groups, CAC bonds and COOR ester groups. Because these composites were biodegradable and biocompatible, it was inferred that they can carry some active compounds (e.g., drugs, enzymes, antioxidants, coenzymes) to be used in medical applications.

Song et al., developed hydrophilic (10 nm) and hydrophobic (5 nm) encapsulated iron oxide nanoparticles (IONPs) into PLA using a one-step water-in-oil-in-water (W/O/W) emulsion process [153]. In their syntheses, it was used commercial PDLLA (M_w_ = 551 kDa, T_g_ = 52.5 °C, LakeShore Biomaterials). As it is known, magnetic nanoparticles have drawn significant attention across fundamental and applied research areas, such as biomedicine and drug delivery. Moreover, the combination of magnetic IONPs (MPIOs) with biodegradable polymers such as PLGA expands their biomedical applicability, for instance, in magnetic resonance imaging (MRI) cell tracking [154]. The authors achieved that the adjustment of emulsification at high temperature (HT = 60 °C) to room temperature (RT = 25 °C) gave raise to PLA-IONPs composite particles from hollow microparticles to solid nanospheres. They also claimed that the fast and facile preparation allows the inclusion of other water-soluble molecules (e.g., penicillin, doxorubicin hydrochloride), as well as potential scale-up.

Lagarrigue et al., prepared functionalized poly(D,L-lactide) (PDLLA) and SiO_2_–CaO binary bioactive glass nanoparticles using the unidirectional freeze-casting method [155]. The authors aimed the obtention of a macroporous composite for bone substitution. They synthesized PDLLA (M_n_ = 8.4 kDa) with fine control of grafting processes of the bioactive glass nanoparticles. Using SEM images, they proposed that PDLLA-grafted nanoparticles are more homogeneously distributed on the scaffolds than the nongrafted ones. They also claimed that this method of fabrication was a new way to achieve bioactive glass/polyester nanocomposite scaffolds, linking the “bricks-and-mortar” concept and freeze-casting. The interparticle interactions and their mechanical properties are highly dependent on the nanoparticle diameter and molar mass of grafted and free PDLLA used for scaffold formulation.

Nerantzaki et al., studied a series of PDLLA nanocomposites with different amounts of silica (2.5–20 wt.%) [156]. The preparation of the polymer nanocomposites (PNCs) of PDLLA/SiO_2_ involved a novel two-step synthesis: ROP of D,L-lactide and polycondensation. Based on the M_n_ values, it was demonstrated that the adaptive time-temperature-step control strategy could be an effective method to make high molar mass PDLLA. They also had experimental strong evidence of a rigid amorphous polymer formation fraction at interfaces with the nanoparticles. There was an increased T_g_ in the nanocomposites, as well as higher thermal stability of PDLLA with the addition of SiO_2_. Thus, these properties confirmed the improvement of PNCs compared to the original matrices of the materials.

**Figure 12:**
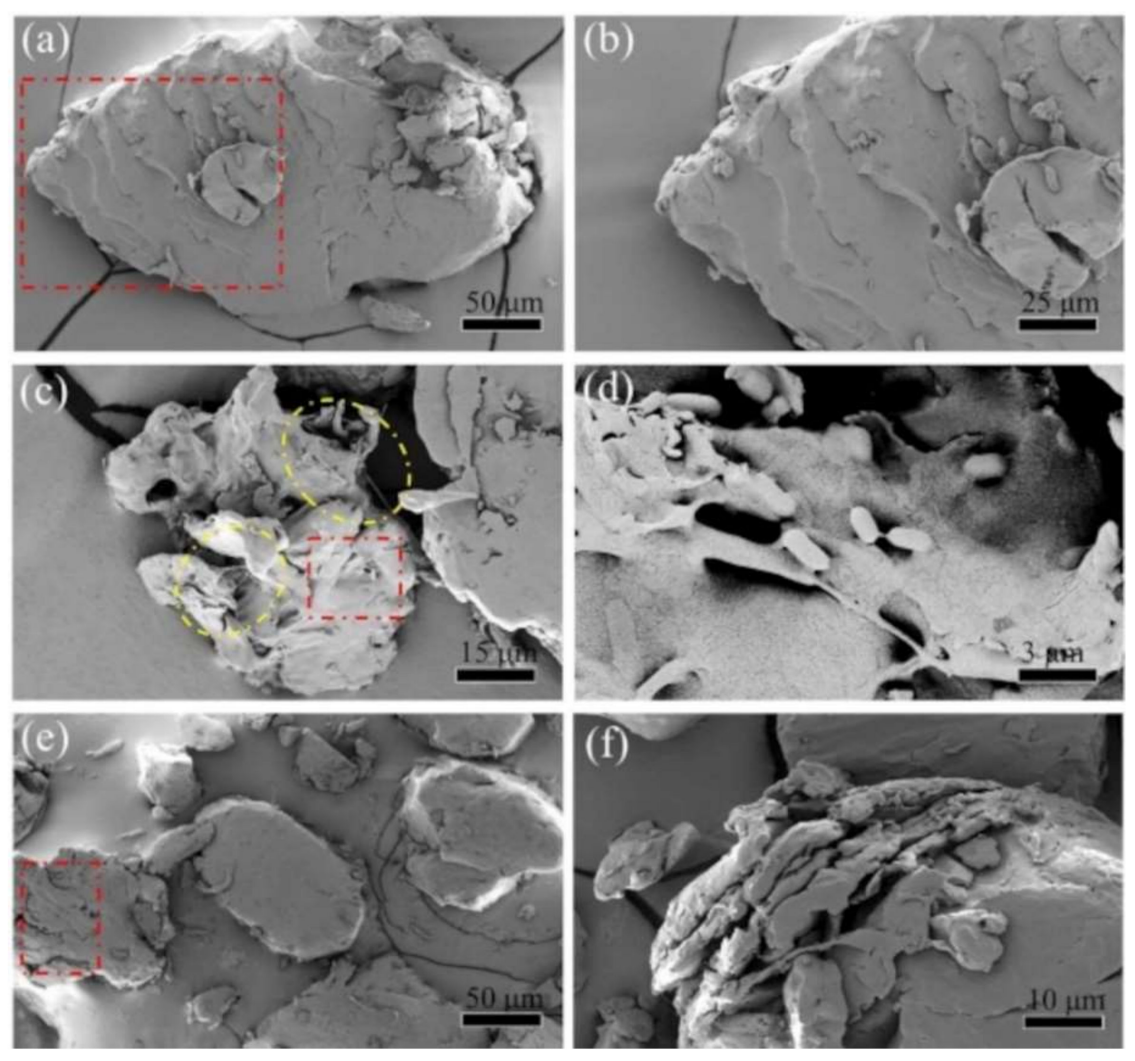
SEM images of sewage incubated PLA MPs before and after removing biofilms, with pristine PLA MPs as control. (**a**) The surface morphology of pristine PLA without any treatments. (**c**) The surface morphology of PLA MPs with incubation time for 35 days. (**e**) The surface morphology of PLA MPs after removal of adherent biofilms. The right graphs (**b**–**f**) exhibited surface morphology of specific areas (marked as yellow or red in (**a**,**c**,**e**), respectively) that were selected and magnified. Obtained from reference [157]. Copyright @ 2022 Elsevier, License Number: 5303830085445.

Sun et al., verified Cu(II) adsorption by poly(lactic acid) microplastics (PLA MPs, NatureWorks, 4230D, density = 1.15 g/cm^3^, purchased from Yitong Plastic Chemical Co., Jinhua, China), which was recovered with biofilms. Biofilms were formed by sewage incubation (Quyang wastewater treatment plant, Shanghai, China) [157]. The PLA MPs showed a high monolayer Cu(II) adsorption mainly due to electrostatic attraction, hydrogen bonding, and surface complexation between copper and functional groups on biofilms. Moreover, the adsorption of Cu(II) was dependent on the pH because of the interaction between the negative charge of MPs and the Cu(II) species. The authors also verified the degradation of PLA MPs under microbial colonization. SEM images shown rod-like bacteria on the surface of the PLA MPs (Figure 12).

Fan et al., analyzed how the hydrolytic degradation of the PDLLA foam gasket was affected by mechanical load [158]. An evaluation of the degradability of the polymer is an important step before clinical application. In this sense, they used commercial PDLLA (molar mass of 200 kDa, Dikang Biomedical Co., LTD, Chengdu, China) for the tests. The authors pointed out that degradable polymers are required to maintain acceptable mechanical properties and suitable degradation rates corresponding with bone healing rates. They concluded that the degradation rates under continuous loads were evidently quicker than those without load and that in vivo PDLLA degradation would not only be influenced by the local solution but also by the surrounding load.

Li et al., studied the toxicity of commercial biodegradable PDLLA films (Changchun SinoBiomaterials, Changchun, China) [159]. Their goal was to develop an anti-adhesion polymer film biomaterial for nerve repair, evaluating its biocompatibility. PDLLA films were used to prepare a solution of PDLLA film extracts (PDLLA/saline = 0.2 g/mL), and these extracts were tested using four concentrations of PDLLA (12.5%, 25%, 50%, and 100%). The novel material showed a porous structure with better mechanical, flexibility and controlled degradation properties, compared to traditional non-porous films. The general results indicated that PDLLA films have excellent biocompatibility and no toxicity in vivo, which potentially place this material to be used in promoting nerve regeneration.

## 6. Conclusions and Perspectives

There are several polymers used in the industry today. Among them, Poly(lactic acid) (PLA) is based on renewable resources and has the potential to be used in sustainable plastics. In order to enhance the attractiveness of PLA, the use of cheaper monomers (e.g., D,L-lactic acid) should be considered. The energy cost for the production of poly(D,L-lactic acid) (PDLLA) is also of great attraction since, in many cases, there is no need to separate the polymer-forming stereoisomers. The production of PDLLA-based on the monomers D,L-lactic acid or rac-lactide leads to the formation of amorphous materials with low crystallinity, but high hydrophobicity. However, properties such as low T_m_ and rapid degradation time (e.g., 3–6 months) open an opportunity to design this biodegradable polymer for many applications in diverse areas. In addition, new research involving the use of different catalysts seeking high selectivity in the synthesis of different PDLLA may enhance the interest of the industry in the production of this material with increasingly specific applications at a lower cost. A few applications in the biomedical area illustrated the various possibilities of the use of PDLLA. Compared with polystyrene, for instance, PDLLA-based materials have the presence of –COOH and –OH groups that can improve the surface chemistry, which may lead to higher interaction with different components. It is known that important properties of PLA-based polymers, such as crystallinity, molar mass, and mechanical strength and toughness are mutually dependent, which associated with other copolymers or blends may create an infinity of tunable materials, as demonstrated in the literature. Thus, the presence of PDLLA in these materials usually granted their biodegradability, which is generally a requisite in biomedical applications. Nevertheless, it is necessary to point out that a significant effort is still required to improve the biodegradability of PLA-containing materials in order to be completely included in a circular economy.

## Data Availability

The data presented in this study are available on request from the corresponding author.

## Abbreviations

BnOH	benzyl alcohol
BTE	bone tissue engineering
CA	cholic acid
CgPCs	chitosan-graft-PDLLA copolymers
CIP	ciprofloxacin
CNCs	cellulose nanocrystals
CT	computerized tomography
CV	cresyl violet
D,L-LA	D,L-lactic acid
DBU	SQ-2 to SQ-6 and 1,8-diazabicyclo[5.4.0]undec-7-ene
DCM	dichloromethane
DESs	Drug-eluting stents
DM	Diabetes mellitus
DMA	Dimethylacetamide
DMAP	4-dimethylaminopyridine
DMC	dimethyl carbonate
DMF	dimethylformamide
DMSO	dimethyl sulfoxide
DP-PDLLA	degree of polymerization of D,L-lactic acid
DSC	Differential Scanning Calorimetry
DS-PDLLA	degree of substitution of PDLLA
DTG	Derivative thermogravimetry
DTX	Docetaxel
EDC/NHS	N-hydroxysuccinimide/N-(3-dimethylaminopropyl)-N′-ethyl-carbodiimide hydrochloride
EDOT	3,4-ethylenedioxythiphene
EG	ethylene glycol
El	elastin peptide
EmimAc	1-ethyl-3-methyimidazolium acetate
ERY	erythromycin
ESC	enantiomorphic site control
FDM	fused deposition modeling
FT-IR	Fourier-transform infrared spectroscopy
GA	glycolic acid
GE	Gelatin
GMA-g-PEO	glycidyl methacrylate-g-poly(ethylene oxide)
GPC	Gel Permeation Chromatography
H_3_PW	12-tungstofosforic acid
hc-PLA	PLA homocrystals
Hedta	ethylenediaminetetraacetic acid
HEMA	2-hydroxyethyl methacrylate
HMW	High molecular weight
IONPs	iron oxide nanoparticles
LA	lactic acid
LAB	lactic acid bacteria
MBL	a-methylene-g-butyrolactone
m-LA	meso-lactide
M_n_	number-average molecular weight
MN	microneedles
MP	microparticles
MPEG-PCLA	monomethoxy-poly(ethylene glycol)-poly(ε-caprolactone-co-D,L-lactide)
MRI	magnetic resonance imaging
MS	microspheres
MSA	methanesulfonic acid
MS-PDLLA	molar substitution of PDLLA
M_w_	weight-average molecular weight
NEt_3_	triethylamine
NNO	diaminophenoxy
NO	nitric oxide
NP	nanoparticles
OLA	oligo lactic acid
PB	prussian blue
PBDE	polybrominated diphenyl ether
PBNPs	prussian blue nanoparticles
PCL	poly(ε-caprolactone)
PDLA	poly(D-lactic acid)
PDLLA	poly(D,L-lactic acid)
PDLLA-b-PNVP	poly(D,L-lactide)-b-poly(N-vinylpyrrolidone)
PDP	phenyl dichlorophosphate
PEDOT	poly(3,4-ethylenedioxythiphene)
PEG	poly(ethylene glycol)
PEO	polyethylene oxide
PET	polyethylene terephthalate
PGPs	calcium phosphate-based glasses particulates
PHB	polyhydroxybutyrate
PLA	poly(lactic acid)
PLA/TPS	PLA-thermoplastic starch
PLA-g-MA	PLA-g-maleic anhydride
PLA-g-TPS	PLA-g-thermoplastic starch
PLEL	(PDLLA-PEG-PDLLA) hydrogel
PLGA	poly(lactic acid-co-glycolic acid)
PLLA	poly(L-lactic acid)
P_m_	meso dyads
PNCs	polymer nanocomposites
PNVP	poly(N-vinylpyrrolidone)
PS	polystyrene
PTMC	poly(trimethylene carbonate)
PVA	poly(vinyl alcohol)
PVPh	poly(vinylphenol)
rac-LA	racemic lactide
RAFT	reversible addition-fragmentation chain transfer
Rapa	Rapamycin
ROP	Ring opening polymerization
sb-PLA	PLA stereoblocks
sc-PLA	PLA stereocomplexes
SEM	scanning electron microscopy
SnCl2	tin(II) chloride dihydrate
SSF	solid-state foaming
SSP	solid-state polycondensation
STVPh	styrene-co-vinyl phenol
TBAC	tetrabutylammonium chloride
TBD	1,5,7-triazabicyclo [4.4.0] dec-5-ene
T_g_	glass transition temperature
TGA	thermogravimetric analysis
THF	tetrahydrofuran
T_m_	melting temperature
TSA	p-toluenesulfonic acid
TUC’s	thiourea-based organocatalysts
WPDLLA	weight content of PDLLA
wt.%	weight percent
XRD	X-ray diffraction
